# Pedigree-based analysis in multi-parental diploid rose populations reveals QTLs for cercospora leaf spot disease resistance

**DOI:** 10.3389/fpls.2022.1082461

**Published:** 2023-01-06

**Authors:** Zena J. Rawandoozi, Ellen L. Young, Stella Kang, Muqing Yan, Seza Noyan, Qiuyi Fu, Tessa Hochhaus, Maad Y. Rawandoozi, Patricia E. Klein, David H. Byrne, Oscar Riera-Lizarazu

**Affiliations:** ^1^ Department of Horticultural Sciences, Texas A&M University, College Station, TX, United States; ^2^ Norman Borlaug Institute for International Agriculture and Development, Texas A&M AgriLife Research, Texas A&M System, College Station, TX, United States

**Keywords:** *rosa*, *Cercospora rosicola*, flexQTL, haplotype, pedigree-based analysis

## Abstract

Cercospora leaf spot (CLS) (*Cercospora rosicola*) is a major fungal disease of roses (*Rosa* sp.) in the southeastern U.S. Developing CLS-resistant cultivars offers a potential solution to reduce pesticide use. Yet, no work has been performed on CLS resistance. This study aimed to identify QTLs and to characterize alleles for resistance to CLS. The study used pedigree-based QTL analysis to dissect the genetic basis of CLS resistance using two multi-parental diploid rose populations (TX2WOB and TX2WSE) evaluated across five years in two Texas locations. A total 38 QTLs were identified across both populations and distributed over all linkage groups. Three QTLs on LG3, LG4, and LG6 were consistently mapped over multiple environments. The LG3 QTL was mapped in a region between 18.9 and 27.8 Mbp on the *Rosa chinensis* genome assembly. This QTL explained 13 to 25% of phenotypic variance. The LG4 QTL detected in the TX2WOB population spanned a 35.2 to 39.7 Mbp region with phenotypic variance explained (PVE) up to 48%. The LG6 QTL detected in the TX2WSE population was localized to 17.9 to 33.6 Mbp interval with PVE up to 36%. Also, this study found multiple degrees of favorable allele effects (*q*-allele) associated with decreasing CLS at major loci. Ancestors ‘OB’, ‘Violette’, and PP-M4-4 were sources of resistance *q*-alleles. These results will aid breeders in parental selection to develop CLS-resistant rose cultivars. Ultimately, high throughput DNA tests that target major loci for CLS could be developed for routine use in a DNA-informed breeding program.

## Introduction

Roses (*Rosa* spp., family Rosaceae) are one of the most important ornamental plants in the world, holding significant economic, cultural and symbolic value (Debener and Byrne, 2014). Roses have been cultivated for more than 5000 years in Europe and China for ornamental, medicinal, food, and perfumery industries (Widrlechner, 1981; Guoliang, 2003; Rusanov et al., 2009). The *Rosa* genus contains more than 150 species with ploidy levels ranging from diploid to decaploid. Garden rose cultivars are generally diploid, triploid, or tetraploid (Zlesak, 2006; Zlesak et al., 2010). Most current cultivars are susceptible to foliage diseases which cause flowers and leaf spotting, necrosis, and eventually, abscission, growth reduction, and plant death (Horst and Cloyd, 2007). Cercospora leaf spot (CLS) is a foliar disease caused by *Rosisphaerella rosicola* Pass, earlier known as *Cercospora rosicola* Pass (Videira et al., 2017), that was first described in 1874 (Davis, 1938). Although CLS is common globally, it has recently become a significant issue in the southeastern United States (Mangandi and Peres, 2018b). Beyond affecting *Rosa*, species of the *Cercospora* genus cause economic losses in soybeans (*C. kikuchii*), corn (*C. zeae- maydis*), sweet beet (*C. beticola*), coffee (*C. coffeicola*), and others (Smith and Gaskill, 1970; Rupe et al., 1982; Souza et al., 2012; Pham et al., 2015).

Plants infected with CLS develop spotting on leaves, chlorosis, and, in severe cases, defoliation. Symptoms of CLS differ from those of a black spot disease (BSD) as the lesions have light necrotic centers, and the lesion margins are smooth as compared to feathery edges of BSD lesions (Mangandi and Peres, 2018a). Like BSD, *R. rosicola* fungal spores are dispersed by water splashing and wind (Dunwell et al., 2014). Environmental conditions such as temperature between 20 to 30°C, high relative humidity, and leaf wetness play a critical role in the accumulation and spread of spores. Other factors reported affecting the growth of CLS are inoculum concentration and light exposure (Cooperman and Jenkins, 1986; Daub and Ehrenshaft, 2000; Daub and Chung, 2007).

Rose fungal diseases are usually controlled by spraying fungicides every 7–14 days when conditions are right for disease development. This could mean 20 or more sprays a year. A survey of rose growers found that disease and pest control costs ranged from $7,000 to $32,000/ha/year in cut flower production (Debener and Byrne, 2014). In addition to the cost of protection, concerns about safety, environmental contamination, and the emergence of pesticide-resistant pathogens/pests have led to developing protocols for integrated pest management (Debener and Byrne, 2014). Key components to more sustainable systems are disease-resistant garden roses. Thus, disease-resistant garden roses are now in high demand from consumers (Harp et al., 2009; Waliczek et al., 2015).

The most challenging issue confronting rose breeders is how to efficiently develop disease-resistant plants, particularly against CLS, BSD, powdery mildew, and rose rosette disease (Byrne, 2015). This is because, in conventional breeding, the selection for disease resistance of these pathogens is a long process and needs two to three field trials as it relies on the natural inoculum in the field. Therefore, marker-assisted selection (MAS) and other genomic approaches are being investigated to help rose breeders make more informed crossing and selection decisions, thus saving time and resources. CLS resistance in roses (*Rosa* ssp.) is reported to be quantitatively inherited with a low to moderate narrow-sense (*h^2^* = 0.51) and moderate to high broad-sense heritabilities (*H^2^* = 0.72) (Kang et al., 2019), indicating that resistance should be a feasible breeding goal. Thus far, no distinct races of CLS or resistance genes have been identified for CLS, whereas genes, QTLs, and pathogenic races associated with BSD (Soufflet-Freslon et al., 2019; Yan et al., 2019; Lopez Arias et al., 2020a; Lopez Arias et al., 2020b) and powdery mildew (Xu et al., 2005; Linde et al., 2006; Xu et al., 2007; Hosseini Moghaddam et al., 2012; Leus et al., 2015) have been reported.

The pedigree-based analysis (PBA) approach (Bink et al., 2012; Bink et al., 2014), which utilizes multiple pedigree-connected families, has been used in various Rosaceous crops. In rose, it has been recently employed to map QTLs associated with black spot disease and rose rosette disease (Yan et al., 2019; Young et al., 2022). Hence, in this study, QTL mapping for CLS resistance through PBA approach will be conducted and followed by haplotype analysis. The ultimate goal of this research is to develop a marker-assisted breeding platform and to develop CLS resistant cultivars. Specifically, this study uses the PBA approach across two sets of diploid rose populations to 1) identify QTLs associated with CLS resistance, 2) identify SNP haplotypes associated with decreased/increased CLS, and 3) estimate QTL genotypes for important rose breeding parents.

## Materials and methods

### Plant materials

Two multi-parental diploid rose populations [TX2WOB (721 individuals) and TX2WSE (378 individuals)] were evaluated under natural inoculum of cercospora leaf spot (CLS) in research fields in two locations in Texas. TX2WOB consists of 11 F_1_ populations evaluated in 2016 and a subset of ten populations of the original population phenotyped in 2019 and 2021 (**Supplementary Table 1**). These populations were derived mainly from *R. wichuraiana* ‘Basye’s Thornless’ (R-Wich) and ‘Old Blush’ (‘OB’) (**Supplementary Figure 1**) (Dong et al., 2017; Yan et al., 2019). The TX2WSE populations composed of six F_1_ rose populations were derived primarily from R-Wich and ‘Srdce Europy’ ('SE') (**Supplementary Figure 2**).

In 2012, one plant of each individual of the TX2WOB populations was planted in the field at the Horticulture Farm at Texas A&M University in College Station, TX, USA (30.63, -96.37) and phenotyped in 2016. In 2018, plants of a subset of ten populations of the original population were planted in a randomized complete block design with two replications (where individual plants were the experimental unit) at the Texas A&M University Horticulture Teaching Research and Extension Center (HortTREC) in Somerville, TX (30.524591, -96.422479) and were phenotyped in 2019 and 2021.

The TX2WSE populations were planted at the HortTREC research plot in 2018 in a completely randomized design with two replications where individual plants were the experimental unit. This multi-parental population was evaluated in three years, 2018, 2020, and 2021(**Supplementary Table 2**). More details on populations and field conditions are described by Rawandoozi et al. (2022).

### Field disease assessment

Cercospora incidence was evaluated by using a percentage-based rating scale of 0 to 9 (0 = no or few cercospora lesions on the plant, 1 = 10% of leaves of the canopy showed lesions, 2 = 20%, 3 = 30%, 4 = 40%, 5 = 50%, 6 = 60%, 7 = 70%, 8 = 80%, 9 = almost all leaves have cercospora lesions). The package `emmeans` v. 1.7.5 of R (v. 4.1.2; R Foundation for Statistical Computing, Vienna, Austria) was used to estimate the least-squares means in all data sets, excluding 2016, to use in the statistical analyses.

All of the 721 individuals of TX2WOB populations were evaluated for CLS in College Station (CS), Texas, during June, Sep., Oct., and Nov., 2016. While 218 and 297 individuals were evaluated in Somerville (SV), TX, from June to Nov. in 2019 and May through Nov. in 2021, respectively (**Supplementary Table 1**). Regarding the TX2WSE populations, all progenies were evaluated for CLS from June through Nov., 2018 and from May to Nov., 2020 and 2021 in SV, TX (**Supplementary Table 2**).

### Heritability, correlation, and genotype by environment interaction

A Shapiro-Wilk test indicated that the CLS data did not fit a normal distribution (*W* ranged from 0.734 to 0.993, *P*< 0.005), except for one data set from the TX2WOB populations (CLS mean SV 2021). Since data transformations did not improve normality, the original data was used as is.

Heritability was estimated using variances calculated from mixed models with a restricted maximum likelihood (REML) estimation method in JMP Pro v. 13.2 (SAS Institute Inc., Cary, NC, USA), with all effects treated as random (Littell et al., 1996). The following model was used:


$$y=\mu +\sigma_{FP}^{2}+\sigma_{MP}^{2}+\sigma_{Progeny(FP,MP)}^{2}+\sigma_{Env}^{2}+\sigma_{FP\times Env}^{2}+\sigma_{MP\times Env}^{2}+\sigma_{Progeny\times Env}^{2}+\sigma_{error}^{2}$$

where μ is the mean; $\sigma_{FP}^{2}$ and $\sigma_{MP}^{2}$ are the female (FP) and male (MP) parent variances, respectively; $\sigma_{Progeny(FP,MP)}^{2}$ is the progeny variance; $\sigma_{Env}^{2}$ is the environmental variance (month/year/location combination); $\sigma_{FP\times Env}^{2}$ , $\sigma_{MP\times Env}^{2}$ , and $\sigma_{Progeny\times Env}^{2}$ are variances due to the interaction of female and male parents and progenies with the year of assessment; and $\sigma_{error}^{2}$ is the error variance.

The sum of the parental variances ( $\sigma_{FP}^{2}$ and $\sigma_{MP}^{2}$ ) was considered as additive variance $\left(\sigma_{A}^{2}\right)$ , progeny variance $\left[\sigma_{Progeny(FP,MP)}^{2}\right]$ was treated as non-additive variance $\left(\sigma_{d}^{2}\right)$ , and the sum of the parental and progeny variances was regarded as the genotypic variance $\left(\sigma_{g}^{2}\right)$ . The interaction of genotype [ $\sigma_{FP}^{2}$ , $\sigma_{MP}^{2}$ , and $\sigma_{Progeny(FP,MP)}^{2}$ ] by environment (year/location) was treated as the genetic-environmental variance $\left(\sigma_{g\times e}^{2}\right)$ . The residual variance, confounded with progeny × environmental variance, was regarded as the error variance $\left(\sigma_{error}^{2}\right)$ .

Broad sense heritability for each set of populations across environments was calculated as: $H^{2}=\frac{\sigma_{g}^{2}}{\sigma_{g}^{2}+\frac{\sigma_{g\times e}^{2}}{E}}$ where *E* indicates the number of environments (years) (Holland et al., 2003; Liang et al., 2017; Wu et al., 2019; Rawandoozi et al., 2021a).

The genotype by environment variance to the genetic variance ratio was estimated as $\sigma_{g\times e}^{2}/\sigma_{g}^{2}$ .

A genotype and genotype-by-environment (GGE) biplot was utilized to display the variation resulting from genotype and genotype by environment interaction (G×E) using the R package ‘GGEbiplots’ v. 0.1.3. Pearson correlation coefficient among environments (years) was calculated.

### Genotyping and consensus map development

Doyle’s CTAB protocol (Doyle and Doyle, 1991) was used to extract genomic DNA using young rose leaves. GBS was performed using the restriction enzyme NgoMIV according to the procedures described by Morishige et al. (2013). Single-end sequencing was accomplished through an Illumina HiSeq 2500 platform. The trimmed reads were aligned to the *Rosa chinensis* v1.0 genome (Hibrand Saint-Oyant et al., 2018) using the CLC Genomics Workbench v9.0 (Qiagen, Boston, MA). After alignment, SNPs were called as described by Yan et al. (2018).

The consensus map for the TX2WOB populations (415 individuals) was developed from five diploid rose populations (**Supplementary Table 3**). The TX2WSE consensus map was created from three diploid rose populations (314 individuals) (**Supplementary Table 4**).

For TX2WOB, before the consensus map development, markers mapped to chromosome 0, non-biallelic markers, and markers missing >10% were removed using Tassel version 5. Then, genotypic data were tested for marker inheritance errors (also known Mendelian-inconsistent errors) using a Microsoft Excel-based tool and custom R scripts. For instance, SNP diplotypes of each progeny were compared with their parental genotypes to detect genotyping errors that were replaced with ‘no call’ if incorrect. Meanwhile, SNP genotypes of the parents were corrected if inconsistent with their progenies. Then, the R package `polymapR` v. 1.1.1 was employed to develop individual population maps, and it was set to perform further filtration to remove duplicated and distorted markers (*P* ≥ 0.001). Further filtration steps were performed to decrease the number of markers to reduce the computation time (e.g., one or two markers were kept at the same genetic position with the priority given to common markers with less missing data). The consensus map was developed using the R package ‘LPmerge’ v. 1.7. The R package ‘LinkageMapView’ v. 2.1.2 and MapChart software v. 2.32 were used to visualize the consensus map. Additional curation in FlexQTL software v. 0.1.0.42 was conducted prior to QTL analysis to identify and fix the singletons and double recombinations based on the ‘SIP_Population_6.csv` and `DoubleRecomb.csv` files. The curation progress was visualized through FlexQTL outputs after each run until all was clear. Further curation for inheritance errors, as determined in the `mconsistency.csv` file of FlexQTL outputs, was conducted. The process was repeated as necessary until no more errors were observed.

As for TX2WSE linkage map construction, the same steps mentioned above were followed, except markers were filtered in PLINK v. 1.9 to zero Mendelian-inconsistent errors per population. For more details on the linkage map development, see Young et al. (2022) and Rawandoozi et al. (2022).

### QTL mapping and characterization

FlexQTL, which implements pedigree-based analysis *via* Markov Chain Monte Carlo (MCMC) Bayesian analysis, was used to perform QTL analysis with data from both multi-parent populations (TX2WOB and TX2WSE). The TX2WOB multi-parent population was genotyped for 1,115 SNP markers and was evaluated for multiple months across three environments (CS 2016, SV 2019, and SV 2021). The TX2WSE population was genotyped for 866 SNP markers and was also evaluated for multiple months over three environments (SV 2018, SV 2020, and SV 2021).

Inference on the number QTLs was based on a pairwise comparison of models (1/0, 2/1, 3/2, and so on) using twice the natural log of the Bayes factor (2lnBF) statistic (Kass and Raftery, 1995). A 2lnBF 0-2 is interpreted as lacking evidence, whereas 2lnBF greater than 2, 5, and 10 are interpreted as positive, strong, and decisive evidence, respectively. The trait was first tested with a mixed model that included QTL with additive and dominance effects. Since a dominance effect was not detected, the analysis was performed with an additive effect model at least two times with variable parameter settings (Verma et al., 2019). MCMC simulation lengths ranged from 100,000 to 800,000 iterations to store a minimum of 1,000 samples with a thinning of 100. The effective sample size (ESS) in the parameter file was set to 101 to ensure sufficient convergence (Bink et al., 2014).

In this study, QTLs were considered significant if the 2lnBF value for QTLs were strong (2lnBF ≥ 5) or decisive evidence (2lnBF ≥ 10) in the same genomic region for most data sets (months) across at least two evaluation years and explained at least 10% of the phenotypic variation. Further analysis was conducted in FlexQTL to re-define QTL intervals in the `MQTRegions.new` file using supporting data files `Post_genome.csv` and `marker map`. The new generated output files e.g., `MQTRRegions.info` was used to recalculate the phenotypic variance explained (PVE) for the discovered QTLs and update information for QTL intensity and interval and mode positions. While some other files (`MQTRegionsGTP.csv` and `mhaplotypes.csv`) were used for haplotype analysis.

From FlexQTL outputs for an additive genetic model, the additive variance $\left(\sigma_{A(trt)}^{2}\right)$ for the trait was obtained from the phenotypic variance $\left(\sigma_{P}^{2}\right)$ minus the residual variance $\left(\sigma_{e}^{2}\right)$ . The PVE for a particular QTL was calculated using the following equation: $PVE_{additive\;\mathrm{mod}el}=\frac{\sigma_{A(qtl)}^{2}}{\sigma_{P}^{2}}\times 100$ where: $\sigma_{A(qtl)}^{2}$ : additive variance of a QTL

The narrow-sense heritability (*h^2^*) was estimated using the following equation:


$$h^{2}=\frac{\sigma_{A(trt)}^{2}}{\sigma_{P}^{2}}$$

QTLs were named according to the QTL naming conventions of the Genome Database for Rosaceae (Jung et al., 2014). For instance, *q*CLS.TX2WOB-LG3.1, *q* stands for QTL, ‘CLS’ is the trait name (cercospora leaf spot), ‘TX2WOB’ or ‘TX2WSE’ stands for the name of the multi-parent population used for construction of the consensus map, ‘LG3’ the linkage group number, and numbers ‘1’ or ‘2’ to differentiate QTLs in the same LG.

Haplotype analysis was performed for SNPs within the region of a major QTL that consistently mapped with either strong or decisive evidence in most environments and showed high PVE. Haplotypes were constructed by using FlexQTL and the `PediHaplotyper` v. 1.0 package of R (Voorrips et al., 2016). Haplotype effects were inferred from combinations of diplotypes. A nonparametric multiple comparison Steel-Dwass test (*P*< 0.05) in JMP Pro v. 13.2 (SAS Institute Inc., Cary, NC, USA) was used to determine the statistical significance of diplotype effects. QTL allele genotypes (*Q* or *q*) were assigned to haplotypes based on the direction of their effects (increasing or decreasing CLS). In the case of a multi-allelic series, *Q*- and *q-*alleles were distinguished by an index number. Lastly, the source of *Q-/q-* alleles was traced back to ancestral origins through pedigree records as described in Rawandoozi et al. (2020; 2021b).

## Results

### Phenotypic data analysis

In the current study, a difference in disease pressure among environments (location/year) was observed. In the TX2WOB populations, data for CLS resistance across three years were skewed towards low scores (**Supplementary Table 5** and **Supplementary Figure 3**). In CS 2016, the lowest CLS score using a 0-9 rating scale was 0.9 in Sep. and the highest was 2.5 in June (**Supplementary Table 5**). No data from CS 2016 exhibited a normal distribution, with disease incidence skewed toward zero (**Supplementary Figure 3A**). In SV 2019, the lowest mean was seen in June (2.2), and the highest (3.7) in Nov. (**Supplementary Table 5**), and all months, except Oct., were normally distributed (**Supplementary Figure 3B**). The lowest mean CLS incidence in SV 2021 was observed in Aug. (1.8), and the highest was in Nov. (2.8). Similarly, most plants this year had low CLS ratings (**Supplementary Figure 3C**).

The CLS resistance in the TX2WSE populations was also skewed towards low scores across three years. Only July and the overall mean of SV 2020 exhibited normal distributions (**Supplementary Table 7** and **Supplementary Figure 4**). The highest disease incidence in SV 2018 was observed in Sep. (1.9) and Oct. (2.0), which may be attributed to high precipitation in these months (**Supplementary Table 6**). In SV 2020, the lowest and highest CLS ratings were observed in Nov. (1.5) and July (4.9), respectively (**Supplementary Table 7**).

In contrast, in SV 2021, the lowest CLS was seen in May (0.6) and the highest observed in June and Nov. (2.8).

### Genotype by environment interactions

In this study, CLS showed high broad-sense heritability (*H^2^*) (0.58 to 0.69) and a moderate G×E variance ratio $\left(\sigma_{g\times e}^{2}/\sigma_{g}^{2}\right)$ (2.13 to 1.33) (**Supplementary Table 8**) in the TX2WOB and TX2WSE populations, respectively. Also, GGE biplot showed that CS 2016 was distinguished from other environments while SV 2019 and SV 2021 similarly discriminated genotypes (**Supplementary Figure 5A**). This result was corroborated by a strong correlation between SV 2019 and SV 2021 (r = 0.94) (**Supplementary Table 9**). However, the longer CS 2016 and SV 2019 vectors indicated genotypes were better discriminated in these environments. Generally, a high to very high positive correlation was observed among years (r = 0.77 to 0.94), supported by the high score for the first principle component (PC1) (90.73%) and the low PC2 score (7.74%).

Regarding TX2WSE, the moderate G×E may have resulted from environmental conditions in SV 2018 and SV 2021, which limited disease development. The CLS incidence scores were mostly low in both years, with a mean ranging from 1.4 in SV 2018 and 1.5 in SV 2021 (**Supplementary Table 7**). Meanwhile, the GGE biplot showed SV 2018 was distinguished from other years (**Supplementary Figure 5B**). This could be due to low incidence combined with young field plots that do not have inoculum well distributed. Also, SV 2020 and SV 2021 similarly discriminated genotypes supported by a strong correlation (r = 0.71) (**Supplementary Table 9**). Overall, moderate to high correlations were found among years (r= 0.55 to 0.71) in data from this population, and PC1 and PC2 values of 87.42% and 3.14%, respectively.

### Consensus map

For TX2WOB, five populations (415 individuals) and nearly 90,502 SNP markers were employed for constructing the integrated consensus map (ICM). The final ICM comprised 4,467 markers with a 6.9 Marker/cM density distributed over 653.1 cM (**Supplementary Table 3**).

For TX2WSE, three populations (314 individuals) with 5,239 to 9,408 markers were used to construct individual linkage maps. The ICM of this population was developed with 2,677 markers and had a length of 758.2 cM with a density of 3.5 markers/cM (**Supplementary Table 4**). Ultimately, there were 398 common markers between the two consensus maps.

After further data curation was conducted through FlexQTL to fix/remove problematic markers and double-recombinant singletons, a total of 1,115 SNP markers for the TX2WOB population and 866 SNP markers for the TX2WSE population were utilized for QTL mapping. A detailed description can be found in Young et al. (2022) and Rawandoozi et al. (2022).

### Genome-wide QTL analysis

Narrow-sense heritability for CLS estimated with FlexQTL ranged from low to moderately high (**Supplementary Table 10** and **11**). In the TX2WOB population, the lowest *h^2^* (0.17) for CLS was observed in July and Nov. in the SV 2021 environment, whereas the highest *h^2^* (0.63) was obtained when a CLS-mean for CS 2016 was used (**Supplementary Table 10**). As for TX2WSE, the lowest *h^2^* (0.27) was found in Aug. and Nov. of SV 2020, and the highest *h^2^* was seen for the CLS-mean of SV 2021 (0.58) (**Supplementary Table 11**).

With FlexQTL, 18 QTLs associated with CLS were mapped on all seven LGs across the three years in the TX2WOB population (**Table 1** and **Supplementary Table 10**, **Supplementary Figure 6**, **7**, and **8**). In the TX2WSE population, 20 QTLs were detected across all LGs over three years (**Table 2** and **Supplementary Table 11**, **Supplementary Figure 9**, **10**, and **11**). Detected QTLs were compared across datasets. If QTL intervals overlapped in the same genomic regions, these were considered to be the same QTL. Also, QTLs that were detected in data from most environments with strong/decisive evidence and showed large effects (PVE%) were considered major QTLs.

**Table 1:** QTL name, linkage group (LG), interval, QTL peak mode (Mode), posterior intensity (QTL intensity), phenotypic variance explained (PVE), and Bayes factor (2lnBF) for the cercospora leaf spot (CLS) evaluated in Texas on 11 rose diploid populations (TX2WOB) across multiple months and overall mean in 2016 in College Station (CS) and on a ten-population subset in 2019 and 2021 in Somerville (SV).

QTL name	Month	Year	LG	Mode	Interval		QTL intensity	PVE	2lnBF
				cM (Mbp)	(cM)	(Mbp)		(%)	
*q*CLS.TX2WOB-LG1	Mean	2019	1	69 (60.56)	[68.0 - 69.8]	[57.86 - 60.95]*	0.45	14	2.1
	Nov.	2021	1	69 (60.56)	[68.0 - 69.8]	[57.86 - 60.95]*	0.53	20	2.6
*q*CLS.TX2WOB-LG2.1	Nov.	2019	2	37 (26.06)	[29.9 - 37.9]	[22.89 - 28.13]	1.09	19	12.1
*q*CLS.TX2WOB-LG2.2	July	2019	2	62 (59.96)	[57.2 - 62.9]	[56.76 - 60.43]*	0.42	9	3.0
*q*CLS.TX2WOB-LG3.1	Mean	2021	3	3 (8.6)	[1.8 - 12.9]	[6.45 - 11.17]	0.75	27	4.6
*q*CLS.TX2WOB-LG3.2	Nov.	2016	3	33 (22.81)	[25.4 - 35.5]	[18.88 - 23.49]*	1.01	13	28.8
	Sep.	2016	3	35 (23.49)	[25.4 - 35.5]	[18.88 - 23.49]*	0.70	8	3.4
	Mean	2016	3	32 (22.1)	[25.4 - 35.5]	[18.88 - 23.49]*	0.98	10	28.9
	Aug.	2019	3	27 (21.4)	[17.2 - 35.5]	[18.49 - 23.49]*	0.78	13	3.8
	May	2021	3	27 (21.4)	[17.2 - 35.5]	[18.49 - 23.49]*	0.52	9	4.4
*q*CLS.TX2WOB-LG3.3	Oct.	2016	3	65 (43.52)	[61.2 - 65.9]	[43.31 - 43.52]	0.68	4	9.3
*q*CLS.TX2WOB-LG4.1	June	2019	4	25 (19.14)	[21.0 - 25.8]	[11.31 - 19.14]*	1.05	8	5.7
*q*CLS.TX2WOB-LG4.2	Sep.	2016	4	36 (39.7)	[35.2 - 36.8]	[35.82 - 39.70]	0.72	33	28.9
	Oct.	2016	4	36 (39.7)	[34.1 - 36.8]	[35.25 - 39.70]	1.01	46	10.8
	Nov.	2016	4	36 (39.7)	[34.1 - 36.8]	[35.25 - 39.70]	1.00	48	25.8
	Mean	2016	4	36 (39.7)	[34.1 - 36.8]	[35.25 - 39.70]	0.94	42	12.6
	Aug.	2019	4	39 (42.64)	[34.1 - 39.9]	[35.25 - 42.64]	0.73	8	3.2
	Mean	2019	4	39 (42.64)	[35.2 - 39.9]	[35.82 - 42.64]	0.80	9	4.3
*q*CLS.TX2WOB-LG4.3	June	2016	4	70 (56.44)	[62.1 - 70.8]	[54.88 - 56.44]	0.98	17	9.7
*q*CLS.TX2WOB-LG4.4	Nov.	2016	4	82 (58.2)	[75.7 - 85.9]	[56.59 - 58.33]	0.77	7	6.2
*q*CLS.TX2WOB-LG5.1	Sep.	2016	5	22 (9.29)	[21.8 - 24.1]	[9.26 - 11.00]	0.76	9	4.5
	Aug.	2021	5	24 (11.00)	[13.3 - 26.7]	[6.38 - 15.11]	0.64	13	3.6
*q*CLS.TX2WOB-LG5.2	Mean	2021	5	87 (84.08)	[86.6 - 91.9]	[84.08 - 85.12]*	0.33	13	2.0
	Oct.	2019	5	107 (85.62)	[86.6 - 108.9]	[84.08 - 85.70]*	0.30	6	2.2
	June	2016	5	108 (85.7)	[91.9 - 108.9]	[85.12 - 85.70]*	1.01	11	7.8
	Mean	2016	5	108 (85.7)	[91.9 - 108.9]	[85.12 - 85.70]*	0.43	12	2.9
*q*CLS.TX2WOB-LG6.1	Oct.	2016	6	4 (1.04)	[0.0 - 5.2]	[0.44 - 2.09]	1.00	18	29.4
	Mean	2016	6	2 (0.94)	[0.0 - 13.0]	[0.44 - 7.84]*	0.50	11	3.1
*q*CLS.TX2WOB-LG6.2	Aug.	2021	6	54 (57.02)	[51.1 - 57.6]	[54.86 - 61.34]	0.63	12	3.1
*q*CLS.TX2WOB-LG7.1	May	2021	7	3 (0.43)	[0.0 - 5.6]	[0.20 - 0.59]*	0.66	13	3.2
	June	2021	7	5 (0.59)	[0.0 - 9.5]	[0.20 - 0.59]*	0.66	13	8.5
*q*CLS.TX2WOB-LG7.2	July	2019	7	31 (12.33)	[29.3 - 32.1]	[12.95 - 13.09]*	1.15	16	7.3
*q*CLS.TX2WOB-LG7.3	Oct.	2016	7	40 (21.51)	[36.0 - 40.1]	[20.05 - 21.51]*	0.65	5	3.1
	July	2021	7	44 (21.78)	[36.0 - 45.9]	[20.04 - 22.64]*	0.54	14	2.6
	June	2021	7	45 (22.64)	[36.0 - 45.9]	[20.04 - 22.64]*	0.67	14	3.2
*q*CLS.TX2WOB-LG7.4	June	2019	7	71 (52.08)	[67.1 - 75.5]	[48.32 - 57.56]	1.08	19	3.5
	Mean	2019	7	71 (52.08)	[69.7 - 73.9]	[50.54 - 55.12]	0.67	24	3.8

*QTL intervals (Mbp) co-localized with TX2WSE population.

**Table 2:** QTL name, linkage group (LG), interval, QTL peak mode (Mode), posterior intensity (QTL intensity), phenotypic variance explained (PVE), and Bayes factor (2lnBF) for the cercospora leaf spot (CLS) evaluated in Texas on six diploid rose populations (TX2WSE) across multiple months in 2018, 2020, and 2021 in Somerville (SV).

QTL name	Month	Year	LG	Mode	Interval		QTL intensity	PVE	2lnBF
				cM (Mbp)	(cM)	(Mbp)		(%)	
*q*CLS.TX2WSE-LG1	Sep.	2018	1	66 (55.82)	[59.70 - 89.40]	[52.10 - 62.81]*	0.78	8	4.3
	May	2021	1	66 (55.82)	[66.51 - 80.27]	[55.82 - 62.77]*	0.87	8	5.5
	Mean	2018	1	84 (62.70)	[80.27 - 89.40]	[62.77 - 62.81]	1.00	11	28.3
	Aug.	2018	1	84 (62.70)	[80.27 - 89.40]	[62.77 - 62.81]	0.77	8	4.1
*q*CLS.TX2WSE-LG2.1	Aug.	2021	2	2 (0.75)	[2.16 - 8.73]	[0.75 - 0.94]	0.42	14	4.3
*q*CLS.TX2WSE-LG2.2	Oct.	2018	2	21 (1.65)	[21.62 - 23.82]	[1.65 - 1.71]	0.73	9	4.4
*q*CLS.TX2WSE-LG2.3	July	2018	2	42 (9.59)	[42.61 - 50.48]	[8.66 - 14.72]	0.84	7	5.5
	June	2018	2	45 (11.41)	[33.15 - 48.01]	[5.57 - 14.86]	0.85	7	3.7
	July	2021	2	50 (14.70)	[48.01 - 56.96]	[14.86 - 26.55]	1.08	6	8.5
*q*CLS.TX2WSE-LG2.4	May	2020	2	80 (60.11)	[79.29 - 84.36]	[56.47 - 60.43]*	0.92	11	9.6
*q*CLS.TX2WSE-LG2.5	May	2021	2	93 (70.88)	[93.04 - 108.81]	[70.88 - 72.31]	1.00	13	12.9
	Mean	2018	2	106 (71.30)	[106.52 - 115.70]	[71.30 - 73.41]	0.85	8	6.9
*q*CLS.TX2WSE-LG3.1	Oct.	2018	3	0 (15.44)	[0.00 - 18.39]	[15.44 - 27.80]*	1.05	9	6.3
	Nov.	2021	3	0 (15.44)	[0.00 - 18.39]	[15.44 - 27.80]*	1.14	13	27.2
	July	2020	3	13 (19.29)	[0.00 - 18.39]	[15.44 - 27.80]*	0.65	13	26.9
	Nov.	2020	3	18 (23.44)	[0.00 - 18.39]	[15.44 - 27.80]*	1.10	10	6.5
	Mean	2020	3	13 (19.29)	[0.00 - 18.39]	[15.44 - 27.80]*	1.15	9	8.8
	Nov.	2018	3	18 (23.44)	[0.00 - 18.39]	[15.44 - 27.80]*	0.96	5	5.0
	Aug.	2020	3	18 (23.44)	[16.31 - 18.39]	[21.51 - 27.80]*	0.60	8	6.0
	Aug.	2018	3	18 (23.44)	[17.21 - 18.39]	[22.90 - 27.80]*	0.87	21	27.7
	July	2018	3	18 (23.44)	[17.21 - 18.39]	[22.90 - 27.80]*	1.07	25	27.9
	Sep.	2018	3	18 (23.44)	[17.21 - 18.39]	[22.90 - 27.80]*	1.04	12	12.8
	Mean	2018	3	18 (23.44)	[17.21 - 18.39]	[22.90 - 27.80]*	1.01	13	28.4
	July	2021	3	18 (23.44)	[17.21 - 18.39]	[22.90 - 27.80]*	1.00	16	26.9
	Mean	2021	3	18 (23.44)	[17.21 - 18.39]	[22.90 - 27.80]*	1.18	11	8.8
	Aug.	2021	3	18 (23.44)	[16.31 - 18.39]	[21.51 - 27.80]*	1.12	12	10.2
	June	2018	3	24 (30.15)	[17.21 - 25.38]	[22.90 - 30.15]*	1.12	15	11.5
	May	2020	3	25 (30.15)	[17.21 - 25.38]	[22.90 - 30.15]*	1.02	8	9.0
	Oct.	2021	3	23 (30.15)	[18.39 - 25.38]	[27.80 - 30.15]	0.50	7	13.5
*q*CLS.TX2WSE-LG3.2	June	2020	3	38 (34.04)	[33.53 - 38.71]	[33.83 - 34.04]	0.90	8	4.1
*q*CLS.TX2WSE-LG4.1	Nov.	2018	4	26 (20.23)	[22.14 - 32.55]	[11.80 – 25.00]*	0.71	7	8.6
	May	2021	4	26 (20.23)	[24.47 - 27.27]	[12.90 - 20.23]*	0.63	17	3.1
*q*CLS.TX2WSE-LG4.2	Sep.	2018	4	56 (46.61)	[53.62 - 64.54]	[46.34 - 51.97]	0.99	11	12.8
	July	2018	4	65 (52.84)	[64.54 - 69.36]	[51.97 - 54.60]	1.14	8	27.9
	Mean	2018	4	68 (54.51)	[61.60 - 69.36]	[49.39 - 54.60]	0.86	9	12.4
	June	2018	4	68 (54.51)	[64.54 - 69.36]	[51.97 - 54.60]	0.93	8	5.4
*q*CLS.TX2WSE-LG5.1	June	2020	5	15 (0.56)	[14.57 - 19.32]	[0.13 - 2.17]	0.78	7	9.9
*q*CLS.TX2WSE-LG5.2	July	2020	5	68 (31.23)	[65.21 - 71.35]	[23.83 - 32.30]	0.99	7	9.7
	Mean	2020	5	68 (31.23)	[62.02 - 71.35]	[24.56 - 32.30]	0.85	5	6.2
*q*CLS.TX2WSE-LG5.3	May	2020	5	99 (67.31)	[93.50 - 100.74]	[63.80 - 70.27]	0.50	9	2.4
	Mean	2018	5	100 (68.85)	[96.65 - 100.74]	[64.55 - 70.27]	0.77	8	3.4
*q*CLS.TX2WSE-LG5.4	Nov.	2018	5	110 (76.77)	[105.96 - 115.92]	[71.56 - 85.07]*	0.78	5	2.5
	Nov.	2021	5	111 (75.88)	[109.14 - 115.92]	[75.03 - 85.07]*	1.09	8	13.6
	Mean	2021	5	114 (78.92)	[111.68 - 119.42]	[75.88 - 85.7]*	1.06	8	27.4
	Oct.	2021	5	123 (85.53)	[115.92 - 123.57]	[85.07 - 85.53]*	0.88	10	27.3
*q*CLS.TX2WSE-LG6.1	June	2018	6	20 (7.17)	[18.92 - 23.26]	[6.78 - 9.26]*	0.51	10	3.7
	July	2021	6	20 (7.17)	[18.92 - 23.26]	[6.78 - 9.26]*	0.58	12	14.0
	Nov.	2018	6	24 (8.94)	[23.26 - 28.67]	[9.26 - 12.08]	1.04	8	10.1
	Mean	2018	6	24 (8.94)	[23.26 - 28.67]	[9.26 - 12.08]	1.00	13	28.4
	Nov.	2021	6	26 (12.04)	[23.26 - 26.66]	[9.26 - 12.04]	1.06	15	28.2
	Oct.	2018	6	28 (12.08)	[23.26 - 28.67]	[9.26 - 12.08]	1.01	7	6.8
*q*CLS.TX2WSE-LG6.2	Mean	2021	6	31 (17.92)	[31.88 - 36.84]	[17.92 - 33.61]	1.11	21	28.1
	Sep.	2018	6	31 (17.92)	[31.88 - 36.84]	[17.92 - 33.61]	0.99	13	9.4
	Aug.	2020	6	34 (29.54)	[31.88 - 36.84]	[17.92 - 33.61]	0.89	17	26.9
	Nov.	2020	6	34 (29.54)	[34.36 - 36.84]	[29.54 - 33.61]	1.24	18	27.5
	Aug.	2018	6	36 (33.61)	[31.88 - 36.84]	[17.92 - 33.61]	0.57	9	8.6
	June	2020	6	36 (33.61)	[34.36 - 36.84]	[29.54 - 33.61]	1.20	17	25.6
	May	2020	6	36 (33.61)	[34.36 - 36.84]	[29.54 - 33.61]	1.03	22	28.0
	July	2020	6	36 (33.61)	[35.24 - 36.84]	[28.56 - 33.61]	1.12	36	27.7
	Mean	2020	6	36 (33.61)	[34.36 - 36.84]	[29.54 - 33.61]	1.01	21	10.0
	June	2021	6	36 (33.61)	[34.36 - 36.84]	[29.54 - 33.61]	1.00	12	26.9
	Oct.	2021	6	40 (39.17)	[31.88 - 41.17]	[17.92 - 40.73]	1.03	10	26.5
*q*CLS.TX2WSE-LG7.1	Nov.	2018	7	8 (0.97)	[6.45 - 19.30]	[0.39 - 1.06]*	0.82	6	3.5
	July	2020	7	8 (0.97)	[6.45 - 13.84]	[0.39 - 0.94]*	1.04	13	10.3
	July	2021	7	15 (1.19)	[6.45 - 17.80]	[0.39 - 1.20]*	0.62	14	25.9
	Nov.	2021	7	15 (1.19)	[6.45 - 17.80]	[0.39 - 1.20]*	0.89	9	27.0
	Mean	2021	7	17 (1.20)	[11.15 - 17.80]	[0.85 - 1.20]	0.86	12	27.0
	Oct.	2018	7	17 (1.20)	[14.45 - 19.30]	[0.22 - 1.06]*	1.36	11	27.2
	Oct.	2021	7	17 (1.20)	[13.84 - 23.50]	[0.94 - 2.00]	0.78	11	9.8
*q*CLS.TX2WSE-LG7.2	July	2021	7	30 (2.84)	[28.74 - 33.96]	[2.83 - 4.47]	0.84	15	25.9
	Sep.	2018	7	41 (9.29)	[28.74 - 41.63]	[2.83 - 9.29]	1.02	7	5.4
*q*CLS.TX2WSE-LG7.3	June	2018	7	45 (12.03)	[43.56 - 52.67]	[10.60 - 15.34]*	0.98	7	11.5
	July	2018	7	48 (14.51)	[43.56 - 48.92]	[10.60 - 14.51]*	0.98	10	10.3
	Mean	2018	7	52 (15.66)	[48.92 - 54.96]	[14.51 - 16.59]	0.94	10	27.9
	May	2021	7	50 (14.87)	[48.92 - 54.96]	[14.51 - 16.59]	0.65	6	4.1
*q*CLS.TX2WSE-LG7.4	June	2021	**7**	62 (24.12)	[57.02 - 62.64]	[20.15 - 24.12]*	0.71	8	6.9

*QTL intervals (Mbp) co-localized with TX2WOB population.

**Figure 1:**
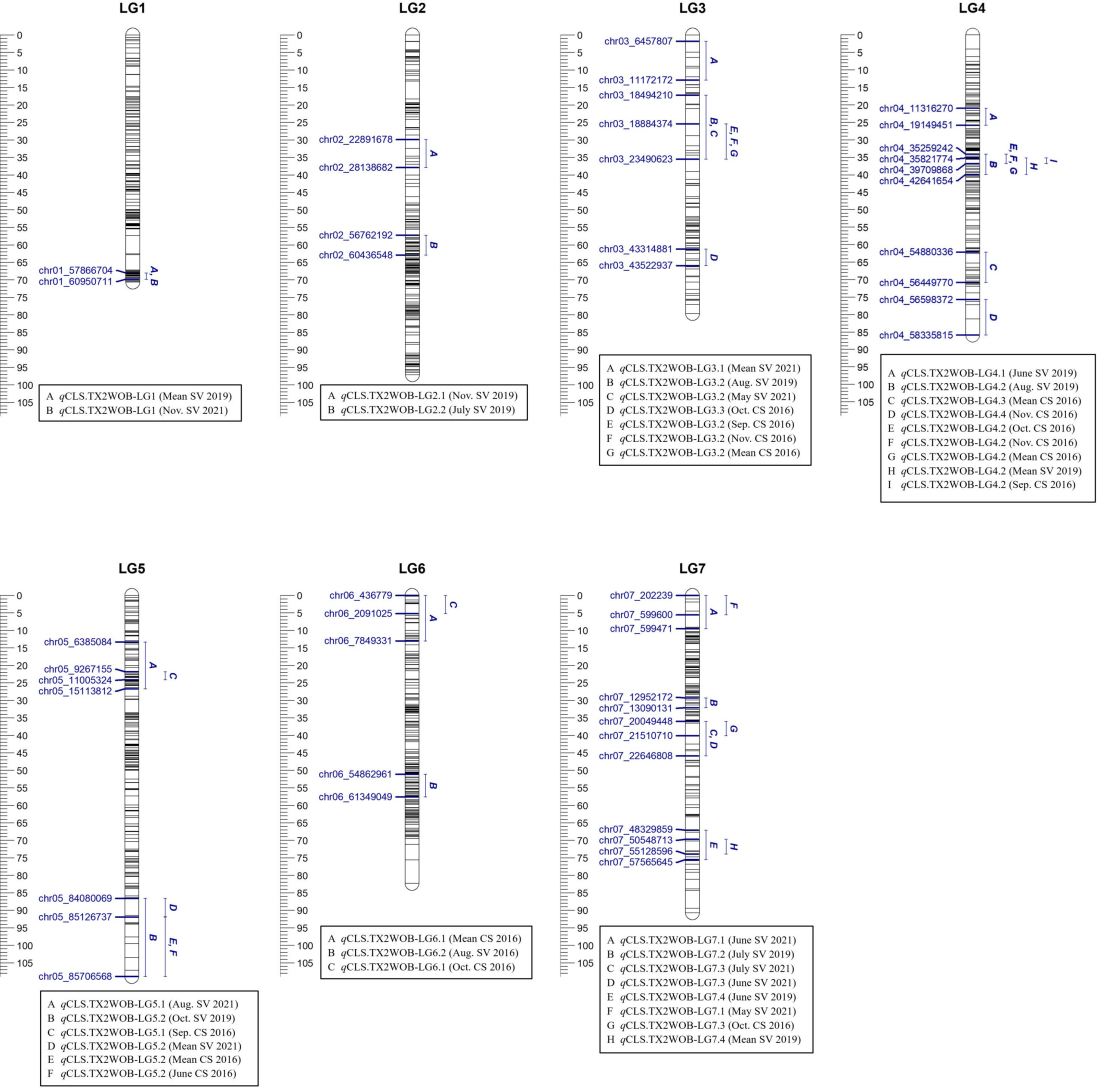
Positions of putative QTLs controlling the cercospora leaf spot disease (CLS) incidence across 11 diploid rose populations at linkage groups (LG) of the five-population (TX2WOB) consensus map. QTL names are listed below each LG. The plot generated using MapChart 2.32.

In the analysis of the TX2WOB population, two major QTLs on LG3 and LG4 were consistently detected at the same positions in data from multiple months across at least two years and showed high PVE (**Table 1**, **Supplementary Table 10**, and **Figure 1**). Therefore, these QTLs were considered for further analysis. *q*CLS.TX2WOB-LG3.2 was identified consistently with either positive or decisive evidence. Peaks for this QTL co-localized across five environments (Sep., Nov. and the overall mean in CS 2016, Aug. 2019, and May 2021 in SV), with QTL peak modes ranging from 27 to 35 cM, and their intervals between 17.2 to 35.5 cM (18.4 - 23.4 Mbp on the rose genome). The proportion of phenotypic variation explained (PVE) by this QTL ranged from 8 to 13% (**Table 1**). The major QTL on LG4, *q*CLS.TX2WOB-LG4.2 was mapped across six environments in CS 2016 and SV 2019 with decisive and positive evidence, respectively. This QTL was clustered at a QTL peak location mode of 36 cM, with an interval between 34.1 and 36.8 cM (35.2 to 39.7 Mbp) in four environments in CS 2016 (except June), and a high posterior intensity and PVE (33 - 48%) (**Table 1**, **Supplementary Table 10**, **Figure 1**, **Supplementary Figure 6**, and **7**). This QTL had wider intervals spanning 34.1 to 39.9 cM (35.2 -42.6 Mbp) in SV 2019 (Aug. and the mean) and had smaller effects (PVE 8-9%).

Four minor QTLs were detected on LGs 1, 5, 6, and 7 (**Table 1**, **Supplementary Table 10**, **Figure 1**, **Supplementary Figures 6**–**8**). One QTL was located at the distal end of LG1, and six QTLs were mapped and clustered at either the proximal or the distal ends of LG5. An additional QTL was located at the proximal end of LG6 and three more minor QTLs were mapped on LG7. The remaining mapped QTLs were environment-specific and detected only in one environment (month).

**Figure 2:**
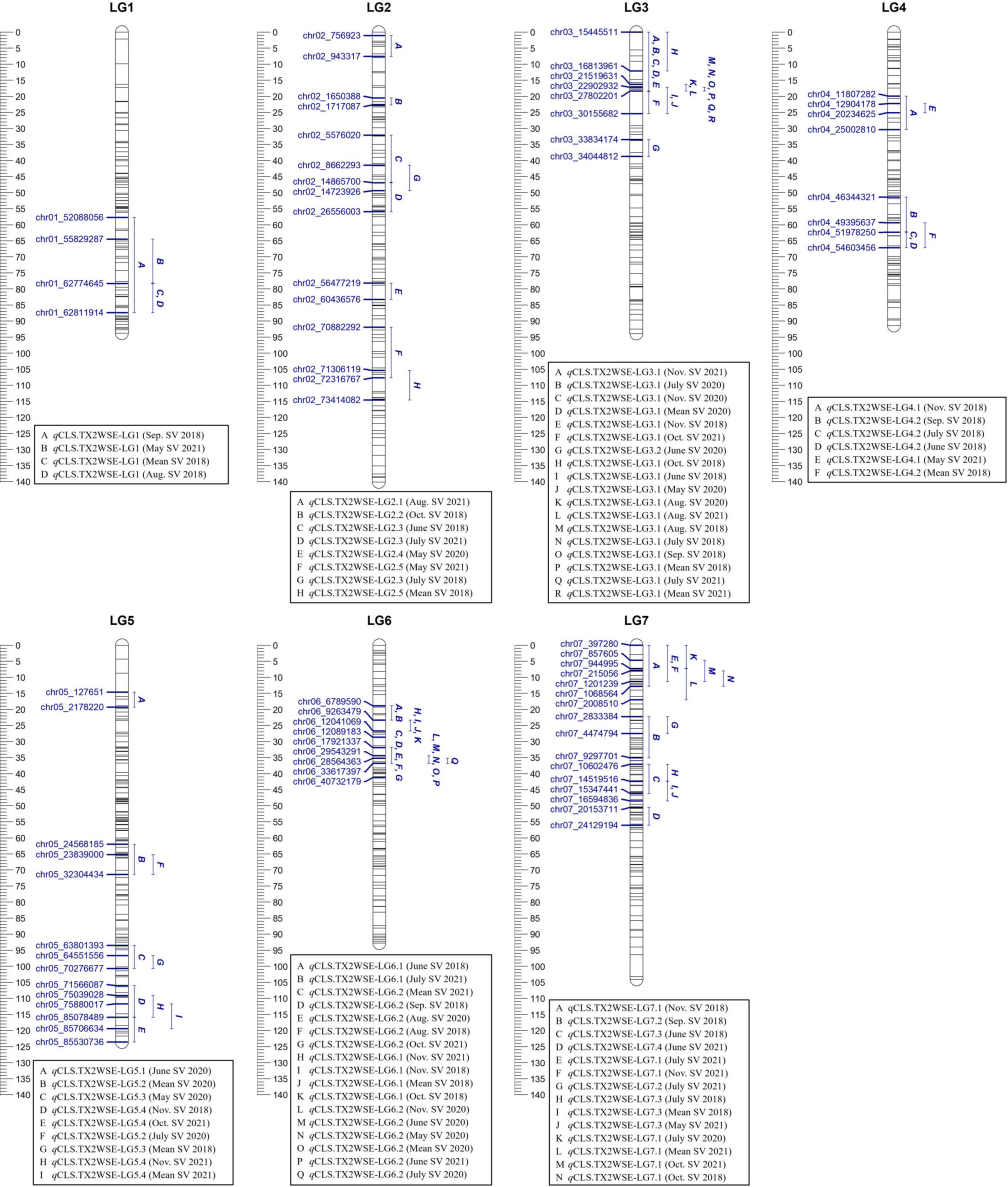
Positions of putative QTLs controlling the cercospora leaf spot disease (CLS) incidence across six diploid rose populations at linkage groups (LG) of the three-population (TX2WSE) consensus map. QTL names are listed below each LG. The plot generated using MapChart 2.32.

Similarly, in the TX2WSE population, 20 QTLs were detected across all linkage groups (**Table 2**, **Supplementary Table 11**, **Figure 2**, **Supplementary Figures 9**–**11**). Two major QTLs on LG3 and LG6 have consistently mapped in 17 and 11 data sets over the three evaluated years, respectively, with high 2lnBF and PVE values. Hence, these QTLs passed our inclusion threshold and underwent downstream analysis.

*q*CLS.TX2WSE-LG3.1 was identified consistently across 17 out of 20 evaluated data sets with either strong or decisive evidence and PVE up to 25%. The intervals of this QTL were variable and overlapped between 0 to 18.39 cM (15.4 to 27.8 Mbp) in six data sets, 16.31 to 18.39 cM (21.5 to 27.8 Mbp) over eight data sets, and 17.21 to 25.38 cM (22.9 to 30.1 Mbp) in three environments (**Table 2**, **Supplementary Table 11**, and **Figure 2**). However, an interval ranging from 16.31 to 18.39 cM was the most common and was supported by the trace plots with high QTL intensity, except for Aug. SV 2020 (**Supplementary Figure 9**, **10**, and **11**). The major QTL *q*CLS.TX2WSE-LG6.2 was common across 11 environments in three years and consistently showed decisive evidence with high posterior intensity, explaining up to 36% of the phenotypic variation (**Table 2**; **Supplementary Table 11**, **Figure 2**; **Supplementary Figures 9**–**11**). The peaks for this QTL were clustered at 31, 34, 36, and 40 cM, however, the peak at 36 cM was the most predominant. The QTL interval ranged from 31.88 to 36.84 cM (17.9 to 33.6 Mbp), excluding Oct. SV 2021, which had a wider interval (31.88 to 41.17 cM).

Several minor QTLs were identified and distributed over all LGs in this population (**Table 2**, **Supplementary Table 11**, **Figure 2**, **Supplementary Figures 9**–**11**). This included one QTL on the distal end of LG1, two on LG2, two on LG4, three on LG5, one on LG6, and three distributed throughout LG7. The rest mapped QTLs were considered environment-specific since they only appeared in one data set.

### QTL genotypes and the interplay between major QTLs

In this study, two QTLs in each population were considered for downstream analysis, including *q*CLS.TX2WOB-LG3.2, *q*CLS.TX2WOB-LG4.2, *q*CLS.TX2WSE-LG3.1, and *q*CLS.TX2WSE-LG6.2. These QTLs were consistently mapped with the highest evidence, intensity, and PVE.

In data from the TX2WOB population, three statistically different QTL genotypes (*qq*, *Qq, QQ*) were predicted at *q*CLS.TX2WOB-LG3.2, where *q* and *Q* were associated with low and high disease incidence, respectively. CLS incidence averaged 3.88, 2.78, and 1.1 for offspring with *QQ*, *Qq*, and *qq*, respectively (**Supplementary Figure 12A**). The QTL genotypes at *q*CLS.TX2WOB-LG4.2 had an average CLS incidence of 5.0 and 1.7 for progenies having the *Qq* and *qq* genotypes, respectively. There were no individuals with the *QQ* genotype class (**Supplementary Figure 12B**). Generally, in this population, unfavorable alleles (*Q*) associated with increasing CLS incidence were less common than those with favorable alleles (*q*). Also, the interplay between *q*CLS.TX2WOB-LG3.2 and *q*CLS.TX2WOB-LG4.2 was studied by examining compound QTL genotypes of these two loci. In general, the highest CLS incidence was noticed in individuals having three copies of *Q-*alleles (*QQ*- homozygous at LG3 and heterozygous-*Qq* at LG4), whereas the lowest susceptibility was seen with four copies of *q*-alleles at two loci. The effect of *QQ*-genotypes at both loci could not be determined due to the lack of this QTL genotype at *q*CLS.TX2WOB-LG4.2.

The analysis revealed the single *Q-*dose at *q*CLS.TX2WOB-LG4.2 increased CLS more than a single *Q-*dose at *q*CLS.TX2WOB-LG3.2 (**Supplementary Figure 13**). Similarly, less disease incidence was noticed in offspring with the *qq* genotype at *q*CLS.TX2WOB-LG4.2 than those at *q*CLS.TX2WOB-LG3.2. This indicates that *q*CLS.TX2WOB-LG4.2 has a larger effect on CLS incidence than *q*CLS.TX2WOB-LG3.2.

With respect to the TX2WSE population, three QTL genotype groups were determined for the peak of *q*CLS.TX2WSE-LG3.1. Progenies with *QQ*, *Qq*, and *qq* genotypes had average CLS incidence of 3.19, 2.15, and 2.09, respectively (**Supplementary Figure 12C**). The QTL genotypes at *q*CLS.TX2WSE-LG6.2 decreased from 3.27 to 2.17 and 1.5 for offspring *QQ, Qq* and *qq*, respectively (**Supplementary Figure 12D**).

Thus, in the TX2WSE population, the frequency of favorable alleles (*q*) associated with decreasing CLS was lower as compared to the TX2WOB population. The comparison between *q*CLS.TX2WSE-LG3.1 and *q*CLS.TX2WSE-LG6.2 revealed that one dose of either *Q* or *q* alleles at the LG6 QTL increased/decreased CLS more than those at the LG3 QTL (**Supplementary Figure 14**). This indicates that *q*CLS.TX2WSE-LG6.2 has a larger effect than *q*CLS.TX2WSE-LG3.1 in CLS resistance.

### Haplotype analysis for important QTLs

**Figure 3:**
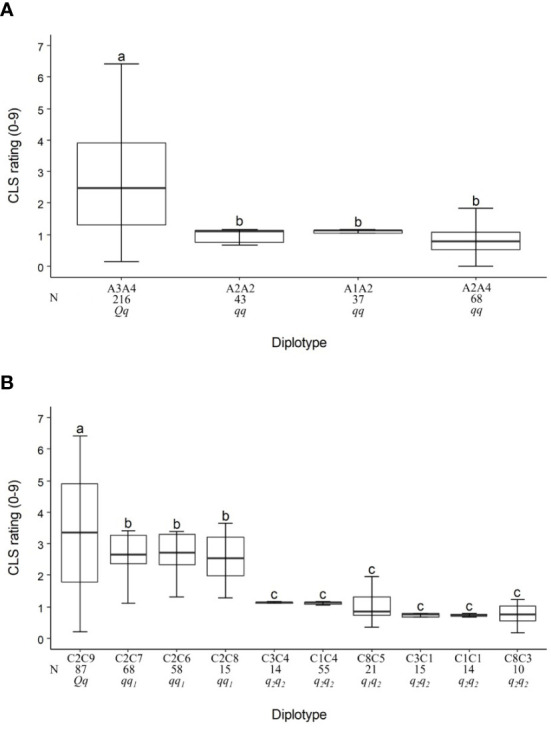
Diplotype effect of the most common haplotypes associated with cercospora leaf spot QTLs *q*CLS.TX2WOB-LG3.2 **(A)** and *q*CLS.TX2WOB-LG4.2 **(B)** in 11 diploid rose populations (TX2WOB). Means not connected by the same letter are significantly different (*P<0.05*) within each linkage group. N = Diplotype sample size.

For the TX2WOB population, the LG3 QTL, *q*CLS.TX2WOB-LG3.2, had four unique haplotypes (A1, A2, A3, and A4) defined with nine SNPs spanning ~10 cM (~4.6 Mbp) across nine parents (**Table 3** and **Supplementary Table 12**). A2 was the high prevalence haplotype (**Figure 3A**). A1, A2, and A4 were associated with a reduction in CLS incidence and were assigned to the *q*-allele, while A3 was the haplotype related to increased disease incidence and assigned to *Q*-allele (**Table 3**). The A3A4 diplotype was present in the highest number of individuals (216) of this population (**Figure 3A**). The estimation of diplotype effects indicated that A3 (*Q*-allele) appeared to lead to greater levels of CLS than A2 (*q*-allele) since the CLS incidence of the A3A4 was higher than for the A2A4. A2 and A4 were of similar magnitude in lowering disease when comparing the diplotype A2A2 to A2A4. The same was true for A2 and A1 when comparing A2A2 to A1A2 diplotypes. So, all diplotypes/haplotypes associated with decreasing CLS showed similar effects (~10%). In general, only A3A4 (*Qq*) showed more disease incidence.

**Table 3:** QTL genotypes of *q*CLS.TX2WOB-LG3.2 and *q*CLS.TX2WOB-LG4.2 for studied breeding parents of the TX2WOB population, with SNP haplotype names, the haplotype’s SNP sequences, and original sources.

LG/interval	Parents	QTL allele		Hap.	SNP haplotype										Successive ancestors
					Allele sequence										founder
LG3 [25.4 - 35.5]	OB	*q*	*♀*	A1	A	A	A	A	C	G	C	T	G		**OB**
	OB	*q*	♂	A2	T	G	C	C	G	T	T	G	A		**OB**
	J3-6	*q*	♂	A2	T	G	C	C	G	T	T	G	A		M4-2 >> **PP-M4-2**
	LC	*q*	*♀*	A2	T	G	C	C	G	T	T	G	A		**LC**
	LC	*q*	♂	A2	T	G	C	C	G	T	T	G	A		**LC**
	VS	*q*	*♀*	A2	T	G	C	C	G	T	T	G	A		**LC**
	VS	*q*	♂	A2	T	G	C	C	G	T	T	G	A		**Violette**
	SC	*q*	*♀*	A2	T	G	C	C	G	T	T	G	A		**LC**
	SC	*q*	♂	A2	T	G	C	C	G	T	T	G	A		**Violette**
	M4-4	*q*	*♀*	A4	A	A	A	A	C	–	–	T	G		WOB26 >> (**R-Wich/OB**)^*^
	M4-4	*q*	♂	A4	A	A	A	A	C	–	–	T	G		**PP-M4-4**
	J14-3	*Q*	♂	A3	A	G	C	C	G	T	T	G	A		**PP-J14-3**
	J14-3	*Q*	*♀*	A3	A	G	C	C	G	T	T	G	A		DD >> **Ducher** or **R-Wich**
	J3-6	*Q*	*♀*	A3	A	G	C	C	G	T	T	G	A		DD >> **Ducher** or **R-Wich**
LG4 [34.1 - 36.8]	OB	*q_2_*	♂	C1	C	T	C	C	A	A	G	G			**OB**
	J3-6	*q_2_*	*♀*	C1	C	T	C	C	A	A	G	G			DD >> **Ducher**
	J4-6	*q*	*♀*	C2	G	A	T	A	A	A	G	G			**R-Wich**
	J14-3	*q*	*♀*	C2	G	A	T	A	A	A	G	G			DD >> **R-Wich**
	J4-6	*q_2_*	♂	C3	G	T	T	A	G	G	T	G			WOB26 >> **OB**
	M4-4	*q_2_*	*♀*	C3	G	T	T	A	G	G	T	G			WOB26 >> **OB**
	OB	*q_2_*	*♀*	C3	G	T	T	A	G	G	T	G			**OB**
	J3-6	*q_2_*	♂	C4	G	A	T	A	A	A	G	–			M4-2 **>> PP-M4-2**
	M4-4	*q_2_*	♂	C5	G	A	–	A	A	A	G	G			**PP-M4-4**
	RF	*q_1_*	*♀*	C6	G	T	T	C	A	A	T	C			**RF**
	RF	*q_1_*	♂	C7	G	T	T	A	A	A	G	G			**RF**
	SC	*q_1_*	♂	C8	G	T	T	C	A	G	G	C			**Violette**
	LC	*q_1_*	*♀*	C8	G	T	T	C	A	G	G	C			**LC**
	SC	*Q*	*♀*	C9	G	T	T	C	A	G	T	G			**LC**
	VS	*Q*	*♀*	C9	G	T	T	C	A	G	T	G			**LC**
	LC	*Q*	♂	C9	G	T	T	C	A	G	T	G			**LC**

^*^Recombination event between two founders *QTL alleles for each parent are presented with ♀ and ♂ for maternal and paternal parent sources, respectively. Allele(s) for predictive SNP marker(s) associated with q-alleles for decreasing CLS are shaded. The identity of the SNP markers and their physical and genetic location is given in **Supplementary Table 12**. Allele(s) for predictive SNP marker(s) associated with q-alleles for decreasing CLS are shaded.

The pedigree map showed that ‘OB’ was the only source of A1 while A2 came from various sources ‘OB’, PP-M4-2, ‘Violette’, or ‘LC’ (**Table 3**). Similarly, A4 of M4-4 appeared to have arisen from recombination events between the parents of WOB26 (‘R-Wich’ and ‘OB’), or it might be inherited from PP-M4-4. Lastly, the source for A3 of parents J14-3 and J3-6 was derived from three distinct sources (PP-J14-3, ‘Ducher’, or ‘R-Wich’).

On LG4, eight SNP markers in *q*CLS.TX2WOB-LG4.2 (34.1 to 36.8 cM) spanning ~2.7 cM were chosen for haplotyping (**Supplementary Table 12**). Nine distinct SNP haplotypes were identified. C2 was the most common haplotype (**Table 3**, **Figure 3**). Haplotypes C1 to C8 were linked to low CLS incidence and assigned to the *q*-allele. C9 was the only haplotype related to increasing disease (*Q*-allele) (**Table 3**). The estimation of diplotype effects indicated that C9 had a larger effect than C6, C7, and C8, since the C2C9 diplotype showed more disease incidence than C2C6, C2C7, and C2C8 (**Figure 3B**). Similar magnitudes in CLS incidence were registered among C6, C7, and C8 based on C2C6, C2C7, C2C8. Both C3 and C1 had an equal effect when comparing C3C4 to C1C4 and C3C1 to C1C1. Likewise, there was no difference observed between C1 and C4 (C3C4 to C3C1 and C1C4 to C1C1) or between C3 and C5 (C8C5 to C8C3). Therefore, there are multiple QTL alleles of different effects at this locus. The haplotype effects order was C9 > [C6 =C7= C8] > [C1=C3=C4=C5] corresponding to *Q, q_1_*, and *q_2_*, respectively. However, the under-representation of some QTL genotypes hindered our ability to conclude the magnitude of the C2 (*q*-allele) effect on decreasing disease.

In this study, some parents shared identical haplotypes even though they were inherited from various ancestors (**Table 3**). For example, the sources of C1 were either ‘OB’ or ‘Ducher’ through J3-6; C2 was inherited from ‘R-Wich’ through J4-6 and J14-3. The haplotypes C3, C4, and C5 were traced back through pedigree to three ancestors, ‘OB’, PP-M4-2, PP-M4-4. Haplotypes C6 and C7 were inherited from ‘RF’, whereas C8 came from ‘Violette’ and ‘LC’, and the latter was the only source for C9.

**Figure 4:**
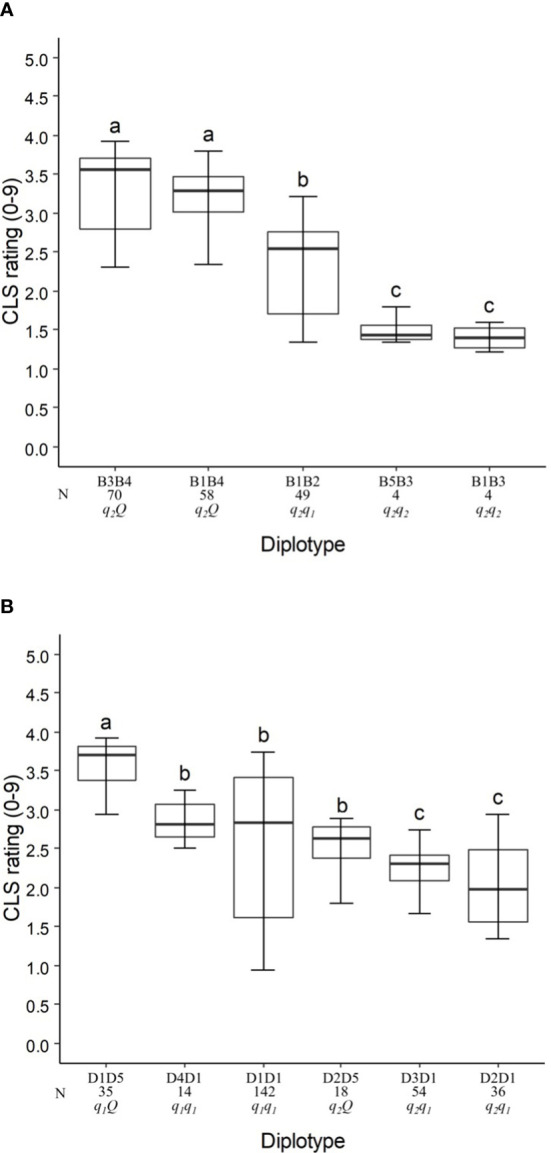
Diplotype effect of the most common haplotypes associated with cercospora leaf spot QTLs *q*CLS.TX2WSE-LG3.1 **(A)** and *q*CLS.TX2WSE-LG6.2 **(B)** in six diploid rose populations (TX2WSE). Means not connected by the same letter are significantly different (*P<0.05*) within each linkage group. N = Diplotype sample size.

In the analysis of the TX2WSE population at *q*CLS.TX2WSE-LG3.1, five distinct SNP haplotypes were identified using six SNP markers (16.31 and 18.39 cM) spanning ~2 cM (**Table 4** and **Supplementary Table 13**). Haplotypes B1, B2, B3, and B5 were linked to lowering CLS incidence, whereas B4 was associated with increasing disease incidence (**Table 4**, **Figure 4A**).

**Table 4:** QTL genotypes of *q*CLS.TX2WSE-LG3.1 and *q*CLS.TX2WSE-LG6.2 for studied breeding parents of the TX2WSE population, with SNP haplotype names, the haplotype’s SNP sequences, and original sources.

LG/interval	Parents	QTL allele		Hap.	SNP haplotype								Successive ancestors
					Allele sequence								(founders in bold)
LG3 [16.31 – 18.39]	M4-4	*q_2_*	*♀*	B1	T	C	T	T	A	G			WOB26 >> **OB**
	M4-4	*q_2_*	♂	B1	T	C	T	T	A	G			**PP-M4-4**
	PH	*q_2_*	*♀*	B1	T	C	T	T	A	G			**OB**
	T7-20	*q_2_*	♂	B1	T	C	T	T	A	G			M4-4 >> WOB26 >> **OB** or M4-4 >> **PP-M4-4**
	T7-30	*q_2_*	♂	B1	T	C	T	T	A	G			M4-4 >> WOB26 >> **OB** or M4-4 >> **PP-M4-4**)
	J14-3	*q_1_*	*♀*	B2	C	C	T	C	A	A			DD >> (**Ducher/R-Wich**)^*^
	SE	*q_1_*	*♀*	B2	C	C	T	C	A	A			**SE**
	SEB-ARE	*q_1_*	*♀*	B2	C	C	T	C	A	A			**SEB-ARE**
	OL	*q_2_*	♂	B3	C	T	A	T	G	A			**R36**
	SEB-ARE	*q_2_*	♂	B3	C	T	A	T	G	A			**SEB-ARE**
	T7-20	*q_2_*	*♀*	B3	C	T	A	T	G	A			SC >> **Violette**
	T7-30	*q_2_*	*♀*	B3	C	T	A	T	G	A			SC >> **Violette**
	PH	*q_2_*	♂	B5	C	T	T	C	A	A			**HIA**
	J14-3	*Q*	♂	B4	C	C	A	T	G	A			**PP-J14-3**
	SE	*Q*	♂	B4	C	C	A	T	G	A			**SE**
	SET-ARE	*Q*	♂	B4	C	C	A	T	G	A			**SET-ARE**
	SET-ARE	*Q*	*♀*	B4	C	C	A	T	G	A			**SET-ARE**
LG6 [31.88 – 36.84]	J14-3	*q_1_*	♂	D1	T	G	A	T	C	C	C		**PP-J14-3**
	PH	*q_1_*	♂	D1	T	G	A	T	C	C	C		**HIA**
	SE	*q_1_*	*♀*	D1	T	G	A	T	C	C	C		**SE**
	T7-20	*q_1_*	*♀*	D1	T	G	A	T	C	C	C		SC >> **Violette**
	T7-30	*q_1_*	*♀*	D1	T	G	A	T	C	C	C		SC >> **Violette**
	M4-4	*q_2_*	*♀*	D2	G	A	G	C	A	T	G		WOB26 >> **OB**
	PH	*q_2_*	*♀*	D2	G	A	G	C	A	T	G		**OB**
	T7-20	*q_2_*	♂	D2	G	A	G	C	A	T	G		M4-4 **>>** WOB26 >> **OB**
	T7-30	*q_2_*	♂	D2	G	A	G	C	A	T	G		M4-4 **>>** WOB26 >> **OB**
	J14-3	*q_2_*	*♀*	D3	T	G	A	T	C	C	G		DD >> **Ducher**
	M4-4	*q_1_*	♂	D4	T	G	A	–	C	C	G		**PP-M4-4**
	OL	*Q*	*♀*	D5	T	A	A	T	C	C	C		**MEV**
	OL	*Q*	♂	D5	T	A	A	T	C	C	C		**R36**
	SE	*Q*	♂	D5	T	A	A	T	C	C	C		**SE**

^*^Recombination event between two founders QTL alleles for each parent are presented with ♀ and ♂ for maternal and paternal parent sources, respectively. Allele(s) for predictive SNP marker(s) associated with *q*-alleles for decreasing CLS are shaded. The identity of the SNP markers and their physical and genetic location are given in **Supplementary Table 13**. Allele(s) for predictive SNP marker(s) associated with q-alleles for decreasing CLS are shaded.

The haplotypes B1, B3, and B5 decreased in a similar magnitude when comparing the B5B3 to B1B3 and B3B4 to B1B4 diplotypes (**Figure 4A**). B3 had a greater effect in lowering CLS than B2 and B4 by comparing B1B2 to B1B3 and B1B3 to B1B4, respectively, and B4 increased CLS relative to B2 (B1B4 to B1B2). Thus, there are three alleles *Q (B4) > q_1_ (B2) > q_2_* (B1, B3, B5) with different effects on disease incidence.

In general, B3B4 and B1B3 showed highest (~35%) and lowest (~20%) CLS incidence, respectively (**Figure 4A**).

The pedigree information revealed that some parents shared the same haplotypes and were inherited from several distinct sources. These were considered identical-by-state (IBS), not identity-by-descent (IBD). B2 was inherited from three sources (IBS), J14-3 derived from recombination between founder haplotypes (‘Ducher’ and ‘R-Wich’), ‘SE’, and SEB-ARE (**Table 4**). Also, the source of B1 were ‘OB’, WOB26, and PPM4-4, while in B3 was inherited from R36, SEB-ARE, and ‘Violette’. Lastly, B4 came from PP-J14-3, ‘SE’, and SET-ARE.

Five SNP haplotypes (D1 to D5) were identified at *q*CLS.TX2WSE-LG6.2 using a total of seven SNPs between 31.88 to 36.84 cM spanning ~5 cM (**Table 4** and **Supplementary Table 13**). Four of these haplotypes, D1 to D4, decreased CLS, and D5 increased disease incidence (**Table 4**). D1 was the most common haplotype, with about 47% of the population (142 individuals) being homozygous for the D1 haplotype (**Figure 4B**).

The haplotype/diplotype effects examination identified that D1 and D4 had a similar effect in decreasing CLS as the D4D1 and D1D1 diplotypes had similar effects (**Figure 4B**). The same was true for D3 and D2 based on D3D1 and D2D1. D1 had a greater effect in lowering CLS than D2 by comparing D1D5 to D2D5. Likewise, D5 had more effect in raising CLS than D1 (D2D5 to D2D1). So, the haplotypes were ordered from higher to lower CLS incidence, D5 > D1, D4 > D3, D2, and were assigned the *Q*, *q_1_*, and *q_2_* QTL alleles, respectively (**Figure 4B**).

D1D5 (*q_1_ Q*) showed the highest CLS (~38%), and D2D1 (*q_1_ q_2_*) had the lowest (~20%) (**Figure 4B**).

The predominant haplotype D1 was inherited from four distinct sources, JPP14-3, HIA, ‘SE’, and ‘Violette’, while D4 came from PP-M4-4, D2 was inherited from ‘OB’ through four distinct parents (M4-4, ‘PH’, T7-20, and T7-30), and D3 came from ‘Ducher’ through J14-3. Lastly, the source of D5 was MEV and R36 through ‘OL’, and ‘SE’ (**Table 4**).

Similar to TX2WOB LG4 QTL, there were two *q*-alleles with different effects on lowering the CLS incidence in this population at both major loci at LG3 and LG6.

## Discussion

### Heritability and G×E

Generally, in this study, the CLS incidence rates were comparable among years in TX2WOB populations, except Sep. CS 2016, which showed less disease pressure, probably due to July’s hot and dry weather (**Supplementary Table 6**) (Weather Underground, 2018). In the TX2WSE populations, the SV 2020 environment showed higher disease incidence than other environments, which could be related to the combination of favorable weather conditions and field plot age. While all data sets of SV 2021 were skewed towards no disease (**Supplementary Figure 4C**), which may be related to lower rainfall and humidity during spring and fall (**Supplementary Table 6**) (Weather Underground, 2018).

In this study, CLS showed low to moderately high *h^2^* and moderately high *H^2^*, as previously reported (Kang et al., 2019). The G×E/G ratios (1.33 to 2.33) and GGE biplots indicated that rose genotypes exhibited different patterns of CLS incidence in different environments (location and/or month/year). Reason for G×E interactions appeared to be the low levels and non-uniform distribution of CLS incidence likely due to unfavorable weather conditions (hot and dry) and/or low initial levels of inoculum, which decreased our ability to distinguish between susceptible and resistant genotypes, as previously reported (Kang et al., 2019). In TX2WOB, the presence of G×E in this population may be attributed to the CS 2016 environment as the total precipitation in this location/year combination was higher than SV 2019 and SV 2021 (~1121 mm vs. 823 and 830 mm, respectively) along with higher relative humidity (**Supplementary Table 6**).

Regarding TX2WSE, the moderate G×E may result from SV 2018 and SV 2021 environments due to the low rate of rainfall and humidity during spring and summer in SV 2018 and throughout SV 2021 (**Supplementary Table 6**) compared to SV 2020, where rain was distributed evenly throughout the growing seasons.

### QTL detection

Using PBA and two diploid rose multiparent populations, 38 QTLs distributed over the seven LGs were identified for CLS resistance, consistent with reports indicating that CLS resistance is polygenic in rose (Kang et al., 2019). Three large-effect QTLs associated with CLS resistance in each population were consistently detected with decisive evidence in most data sets on LGs 3, 4, and 6.

The LG3 QTL was common to both populations and was consistently detected in different years, indicating that this QTL was less affected by environmental factors. The coincidence in the location of these LG3 QTLs (*q*CLS.TX2WOB-LG3.2 and *q*CLS.TX2WSE-LG3.1) suggests that these may be the same QTL. Furthermore, haplotype analysis indicated that the physical positions of the two predictive SNP markers, associated with haplotypes associated with decreasing/increasing CLS, coincided for both LG3 QTL. Also, peaks of these QTLs were clustered at the region between 21.40 and 23.49 Mbp.

The major QTL on LG4 (*q*CLS.TX2WOB-LG4.2) was only detected in the TX2WOB population in the CS 2016 and SV 2019 environments. The moderate G×E/G ratio (2.13) that was observed in this population could be among various factors that prevented this QTL from being detected in data from SV 2021. Other factors include more limited initial inoculum and the age of the field plot.

The LG6 QTL (*q*CLS.TX2WSE-LG6.2) between 17.9 to 33.6 Mbp was specific to the TX2WSE population and was little affected by environmental factors as it was consistently detected over three years. This finding was consistent with the lower G×E/G ratio (1.33) observed in this population compared to TX2WOB (2.13).

We can conclude in this study that using the two large and diverse diploid rose multi-parental populations through PBA facilitated the detection of numerous QTLs with major and minor effects associated with CLS resistance. PBA has been used successfully for quantitative and complex traits on highly heterozygous, clonally propagated crops, including rose (Mangandi et al., 2017; Cai et al., 2018; Rawandoozi et al., 2020; Rawandoozi et al., 2022; Young et al., 2022).

The variability in the number/position of detected QTLs in some data sets in this study was anticipated. This may have resulted from the differences between the consensus maps and/or the disease pressure and environmental conditions that cause the G×E interaction (Kang et al., 2019).

The results showed that *q*CLS.TX2WOB-LG3.2 and *q*CLS.TX2WSE-LG6.2 behaved additively as the phenotypic value of *Qq* genotype class was the mid value between *QQ* and *qq* classes, supported by visual inspection of the mixed model plots, which estimated additive effects. The determination of gene action for *q*CLS.TX2WSE-LG3.1and *q*CLS.TX2WOB-LG4.2 was hampered by the low or lack of representation of progenies having the *qq* or *QQ* genotypic classes. Hence, future QTL mapping studies need to use germplasm of wider diversity to improve the representation of QTL genotype classes/diplotype combinations.

A study by Lopez Arias et al. (2020a) scanned protein sequences from the *Rosa chinensis* (Hibrand Saint-Oyant et al., 2018) genome for the R-gene-related domains. Several identified nucleotide-binding leucine-rich repeat (NBS-LRR) proteins, key initiators of plant defense responses, were located within the genomic regions of the mapped QTLs for CLS resistance.

The LG3 interval included genes involved in response to fungal infection. These genes encode an EMSY-LIKE 1 protein for downy mildew (*Hyaloperonospora parasitica*) resistance in *Arabidopsis* (Tsuchiya and Eulgem, 2011) and cytochromes P450 monooxygenases which are known to be involved in plant defense mechanisms (Schuler and Werck-Reichhart, 2003; Schuler et al., 2006). Pathogenesis-related (PR) thaumatin genes induced by plants in response to different biotic and abiotic stresses also were located close to the LG3 QTL region (Zhang et al., 2018). WRKY (a plant-specific transcription factor) was also found to overlap with this QTL which is known to play vital roles in fungal pathogen defense (Lui et al., 2017). Hence, additional studies focusing on fine mapping of the major loci conferring CLS resistance in this study are necessary to identify candidate genes responsible for this disease.

### Haplotype characterization of significant QTLs

Haplotype characterization indicated the presence of multiple QTL alleles of various effects associated with decreasing CLS incidence for *q*CLS.TX2WSE-LG3.1, *q*CLS.TX2WOB-LG4.2, and *q*CLS.TX2WSE-LG6.2. Differences in the magnitude of the CLS-resistant effect at these loci raise the possibility of multiple functional resistance alleles in these studied populations. QTL*-*alleles with different effects were also described in other studies (Verma et al., 2019; Rawandoozi et al., 2020; Rawandoozi et al., 2021b).

According to pedigree information of these studied populations, ‘OB’, ‘Violette’, and PPM4-4 were common sources of favorable *q* **-**alleles for major loci. In contrast, the sources for unfavorable *Q* **-**alleles were PP-J14-3 (*q*CLS.TX2WOB-LG3.2 and *q*CLS.TX2WSE-LG3.1), ‘LC’ (*q*CLS.TX2WOB-LG4.2), and MEV and R36 (*q*CLS.TX2WSE-LG6.2). Therefore, future selection for either haplotype associated with *q-*allele such as A1, A2, A4, C3, C5, C8, B1, B3, D1, D2, and D4, or against haplotype associated with *Q-*allele such as A3, C9, B4, and D5 might be useful to develop rose populations with a lower CLS incidence.

### Co-localization between CLS and BSD QTLs

The position of CLS QTLs coincided with the positions of BSD QTLs previously reported in similar and different germplasm (Lopez Arias et al., 2020a; Lopez Arias et al., 2020b; Rawandoozi et al., 2022). Thus, these results indicate that there is a relationship between some loci that affect resistance to these two fungal diseases. The major resistance QTL on LG3 for CLS and BSD co-localized in both populations. The QTL from data from the TX2WOB population, *q*CLS.TX2WOB-LG3.2 and *q*BSD.TX2WOB-LG3.2, clustered between 18.8 to 23.4 Mbp. Likewise, the major QTL on LG3 for both diseases in data from the TX2WSE population (*q*CLS.TX2WSE-LG3.1 and *q*BSD.TX2WSE-LG3.1) overlapped between 15.4 to 27.8 Mbp. This specific genomic region on LG3 was previously reported to be associated with BSD using different populations derived from *R. wichurana* (Lopez Arias et al., 2020a; Lopez Arias et al., 2020b).

The haplotypes for the major CLS QTLs on LG3 (*q*CLS.TX2WOB-LG3.2 and *q*CLS.TX2WSE-LG3.1) compared with those earlier reported for BSD revealed that the same predictive markers were simultaneously linked to the CLS resistance allele and the BSD susceptibility allele. This QTL in the TX2WOB population seems to have a greater effect on decreasing CLS than increasing BSD resistance (~28% vs. ~13%). However, different magnitude of effect was observed in the TX2WSE population (~10% vs. ~25%). Thus, this finding implies that LG3 QTL has opposite effects on these two traits. This was supported by a negative correlation (r = -0.30, *P*<0.01) (data not shown) between these traits in this and previous work (Kang et al., 2019) and a report that cultivars with lower BSD incidence showed higher susceptibility to CLS (Hagan et al., 2005).

Moreover, co-localization between minor QTLs for CLS and BSD resistance was also found on LG3, in two regions on LG4, on the proximal and distal ends of LG5 (Lopez Arias et al., 2020a; Lopez Arias et al., 2020b; Rawandoozi et al., 2022), on LG6 QTL and on three regions on LG7 on the upper, middle, and lower parts of LG in TX2WOB.

Consequently, selecting for the CLS resistance QTL on LG3 may be accompanied by BSD susceptibility. In the short term, given that the relative effect of the LG4 and LG6 QTLs were greater than the LG3 QTL in the two populations (~33% vs. ~27% in TX2WOB and ~17% vs. ~10% in TX2WSE), using predictive SNP markers on LG4 and LG6 may be an alternative to breed roses for lower CLS susceptibility. Thus, the genetic information of estimated diplotype effects of this study, such as C3C4, C1C4, C8C5, C3C1, C1C1, C8C3, D3D1, and D2D1, would have a potential advantage in decreasing CLS incidence in rose breeding.

Further investigation on the genetic basis of both diseases is needed using broader and more diverse germplasm evaluated in multiple environments to give deeper insight into the interplay between CLS and BSD. Since both fungal diseases share similar symptoms, which may lead to inaccurate field assessments (Whitaker and Hokanson, 2009b; Whitaker and Hokanson, 2009a), particularly at the early stage of disease development (Horst and Cloyd, 2007), it is recommended to evaluate the disease during late fall in at least a second or third year established field for better disease pressure build-up, and ease of distinguishing between two diseases, and subsequently more accurate phenotyping.

## Conclusion

In this research, for the first time, multiple QTLs with major and minor effects associated with CLS resistance have been reported in rose using QTL mapping through PBA on two multi-parental populations evaluated over five years in two locations in Texas. One major QTL on LG3 was consistently detected with decisive evidence across populations between 18.8 to 27.8 Mbp and explained up to 25% of the CLS phenotypic variation. Two other major QTLs on LG4 (35.8 to 39.7 Mbp, PVE up to 48%) and LG6 (17.9 to 33.6 Mbp PVE up to 36%) were population-specific to TX2WOB and TX2WSE populations, respectively. Several QTLs with minor effects were distributed over LGs 1, 2, 4, 5, and 7. The interplay between the two important QTLs for each population revealed that the LG4 and LG6 QTLs in TX2WOB and TX2WSE, respectively, showed larger effects than the LG3 QTL. Also, this study found multiple *q*-alleles of different effects on major loci. ‘OB’, ‘Violette’, and PP-M4-4 were the sources of *q*-alleles across three loci in both populations. Also, our results from LG3 QTL suggest the existence of negative relationships between CLS and BSD resistance.

In summary, the identification of 38 QTLs for CLS and SNP markers associated with QTL-alleles of the major QTLs and their sources are primary information for rose breeders and a step toward the deployment of DNA-informed techniques to facilitate the selection of new rose cultivars resistant to this disease.

## Data availability statement

The datasets presented in this study can be found in online repositories. The names of the repository/repositories and accession number(s) can be found below: Genome Database for Rosaceae, tfGDR1064.

## Author contributions

DB conceived and designed this study. ZR and MR performed QTL and haplotype analyses. EY, SK, MY, SN, TH, and QF collected and provided phenotypic data. MY and EY collected tissues and extracted DNA. PK conducted genotyping-by-sequencing and SNP calling. ZR and EY curated genotypic data and produced the linkage maps. ZR, DB, and OR-L wrote the manuscript. ZR, EY, SK, MY, SN, QF, TH, MR, PK, DB, and OR-L reviewed the manuscript DB and OR-L provided project supervision.

## Supplementary material

Supplementary Figure 1 Pedigree of the TX2WOB multi-parental population composed of 11 F1 diploid rose populations derived from intercrossing nine genotypes. Red and blue lines link progeny to female and male parents, respectively, generated using PediMap 1.2.

Supplementary Figure 2 Pedigree of the TX2WSE multi-parental population composed of six F1 diploid rose populations derived from nine parents. Red and blue lines link progeny to female and male parents, respectively, generated using PediMap 1.2.

Supplementary Figure 3 Histograms for cercospora leaf spot disease (CLS) incidence for the TX2WOB diploid rose population evaluated in Texas in College Station (CS) 2016 (A), and in 2019 (B), and 2021 (C) in Somerville (SV).

Supplementary Figure 4 Histograms for cercospora leaf spot disease (CLS) incidence for the TX2WSE diploid rose population evaluated in Texas in 2018 (A), 2020 (B), and 2021 (C) in Somerville (SV).

Supplementary Figure 5 Genotype plus genotype × environment (GGE) biplot representing the discrimination and representativeness of environments for the cercospora leaf spot disease (CLS) incidence in Texas College Station (CS) in 2016, Somerville (SV) in 2019 and 2021 for TX2WOB population (A), and SV in 2018, 2020, and 2021 for TX2WSE population (B).

Supplementary Figure 6 Posterior positions (left) and trace samples QTL positions (right) based on an additive model performed using Visual FlexQTL software for cercospora leaf spot incidence in June, Sep., Oct., Nov., and the overall mean in 2016 for 11 diploid rose populations (TX2WOB) in College Station, Texas.

Supplementary Figure 7 Posterior positions (left) and trace samples QTL positions (right) based on an additive model performed using Visual FlexQTL software for cercospora leaf spot incidence in June, July, Aug., Oct., Nov., and the overall mean in 2019 for 10 diploid rose populations (TX2WOB) in Somerville, Texas.

Supplementary Figure 8 Posterior positions (left) and trace samples QTL positions (right) based on an additive model performed using Visual FlexQTL software for cercospora leaf spot incidence in May, June, July, Aug., Nov., and the overall mean in 2021 for 10 diploid rose populations (TX2WOB) in Somerville, Texas.

Supplementary Figure 9 Posterior positions (left) and trace samples QTL positions (right) based on an additive model performed using Visual FlexQTL software for cercospora leaf spot incidence in June, July, Aug., Sep., Oct., Nov., and the overall mean in 2018 for six diploid rose populations (TX2WSE) in Somerville, Texas

Supplementary Figure 10 Posterior positions (left) and trace samples QTL positions (right) based on an additive model performed using Visual FlexQTL software for cercospora leaf spot incidence in May, June, July, Aug., Nov., and the overall mean in 2020 for six diploid rose populations (TX2WSE) in Somerville, Texas.

Supplementary Figure 11 Posterior positions (left) and trace samples QTL positions (right) based on an additive model performed using Visual FlexQTL software for cercospora leaf spot incidence in May, June, July, Aug., Oct., Nov., and the overall mean in 2021 for six diploid rose populations (TX2WSE) in Somerville, Texas.

Supplementary Figure 12 Probable QTL genotype at the signal peak from all progenies for cercospora leaf spot QTLs of diploid rose populations qCLS.TX2WOB-LG3.2 (A) and qCLS.TX2WOB-LG4.2 (B) for TX2WOB and qCLS.TX2WSE-LG3.1 (C) and qCLS.TX2WSE-LG6.2 (D) for TX2WSE.

Supplementary Figure 13 Analysis of the compound QTL genotypes from qCLS.TX2WOB-LG3.2 and qCLS.TX2WOB-LG4.2 on cercospora leaf spot disease rating from all progenies in 11 diploid rose populations (TX2WOB).

Supplementary Figure 14 Analysis of the compound QTL genotypes from qCLS.TX2WSE-LG3.1 and qCLS.TX2WSE-LG6.2 on cercospora leaf spot disease rating from all progenies in six diploid rose populations (TX2WSE).
