# Supplementary figures and images for: Pedigree-based analysis in multi-parental diploid rose populations reveals QTLs for cercospora leaf spot disease resistance
Source: Front Plant Sci. 2023 Jan 6;13:1082461. doi: 10.3389/fpls.2022.1082461 (PMC9859674; doi:10.3389/fpls.2022.1082461)

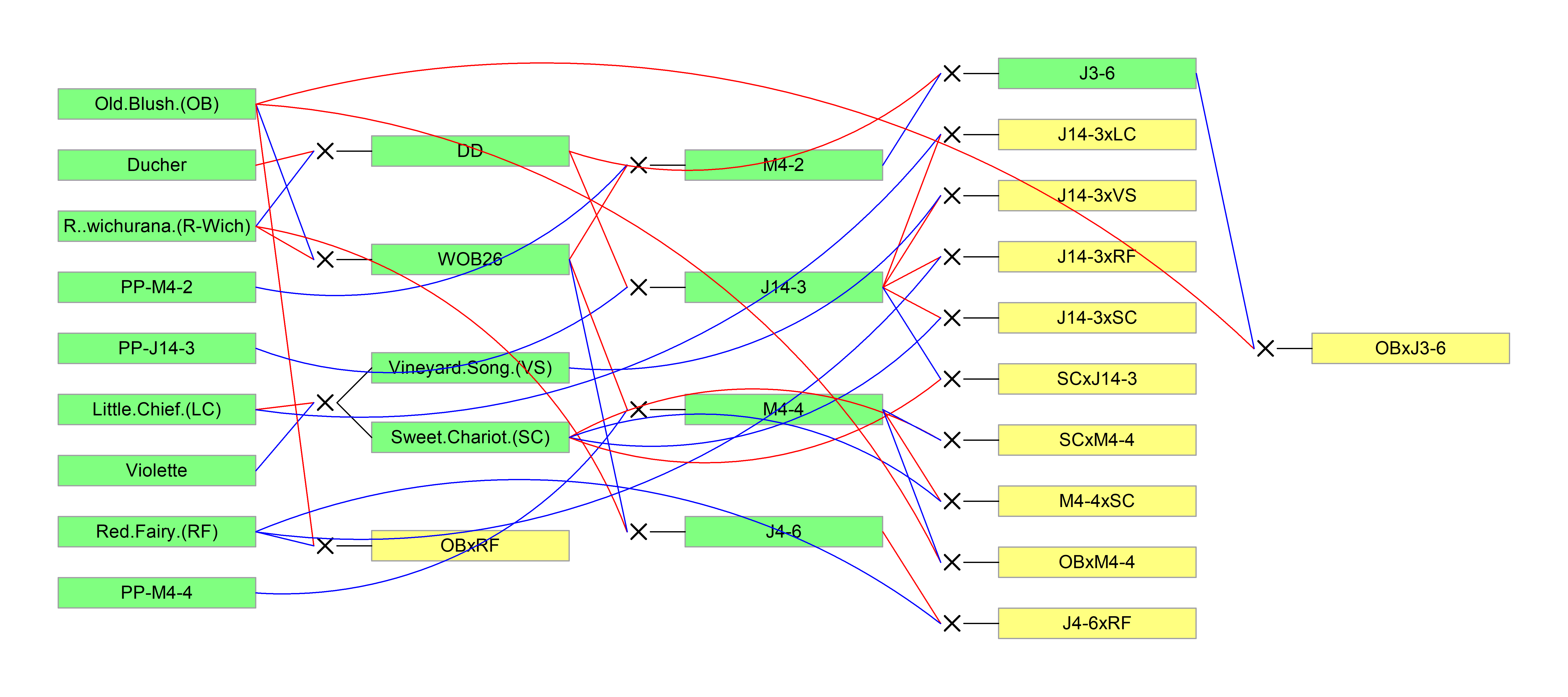

Supplement: Supplementary Figure 1 — Pedigree of the TX2WOB multi-parental population composed of 11 F1 diploid rose populations derived from intercrossing nine genotypes. Red and blue lines link progeny to female and male parents, respectively, generated using PediMap 1.2. [file Image_1.png]

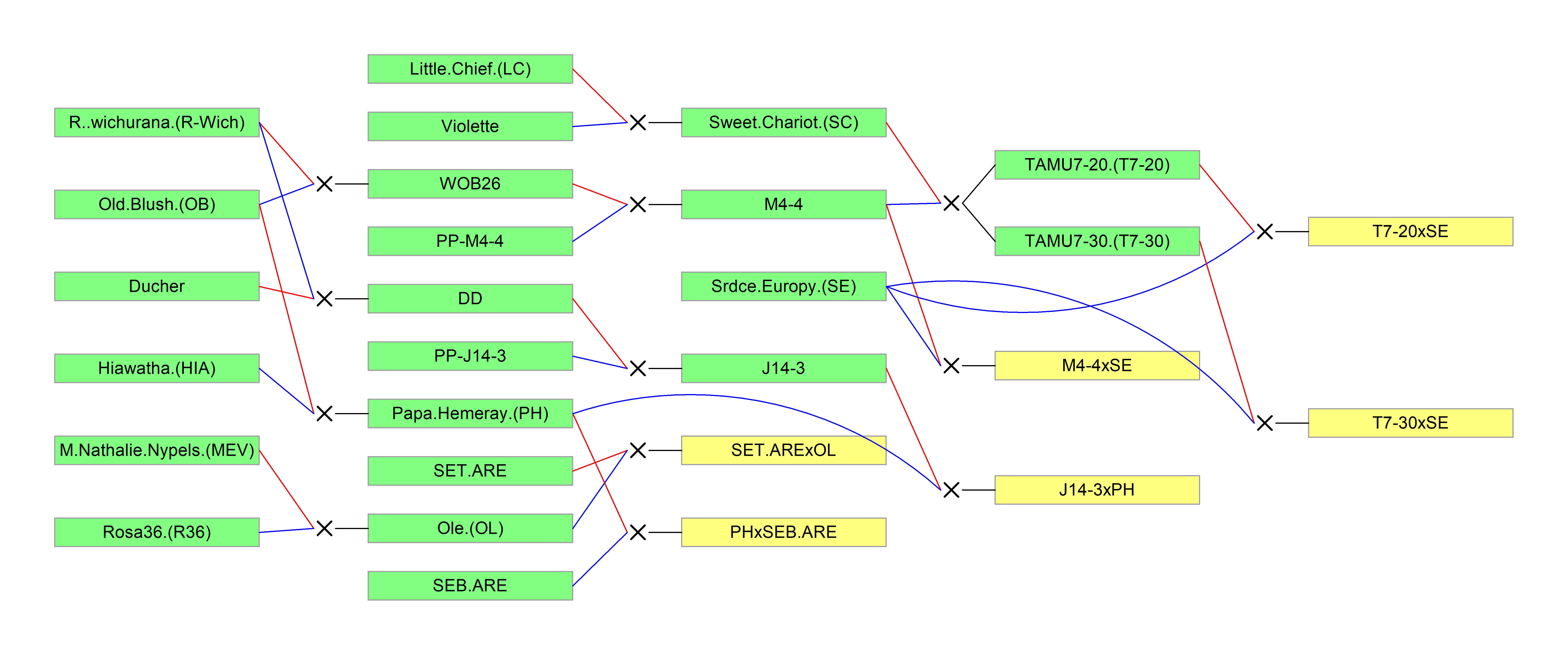

Supplement: Supplementary Figure 2 — Pedigree of the TX2WSE multi-parental population composed of six F1 diploid rose populations derived from nine parents. Red and blue lines link progeny to female and male parents, respectively, generated using PediMap 1.2. [file Image_2.png]

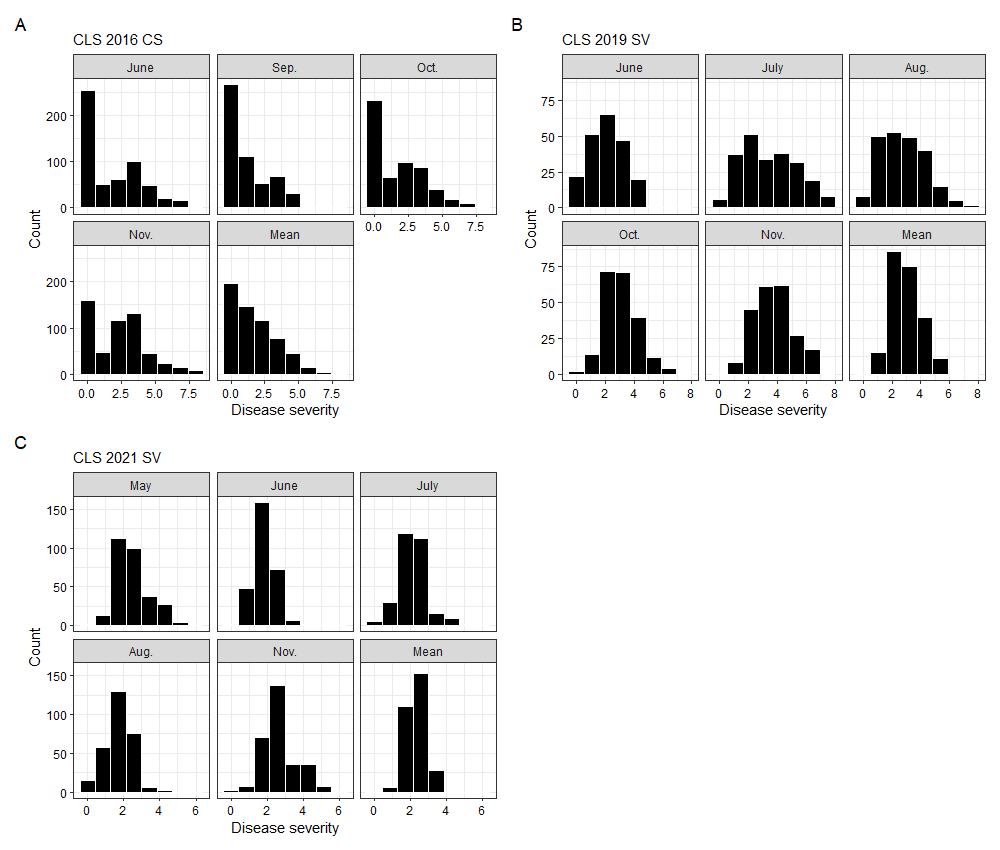

Supplement: Supplementary Figure 3 — Histograms for cercospora leaf spot disease (CLS) incidence for the TX2WOB diploid rose population evaluated in Texas in College Station (CS) 2016 (A), and in 2019 (B), and 2021 (C) in Somerville (SV). [file Image_3.png]

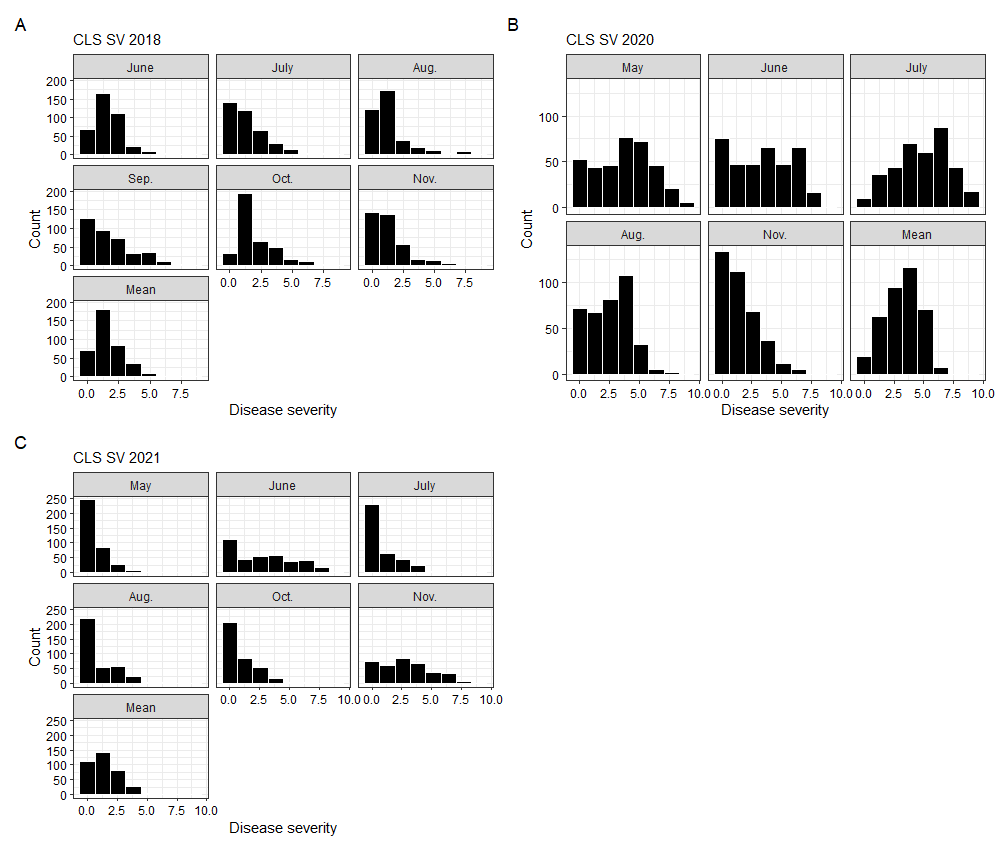

Supplement: Supplementary Figure 4 — Histograms for cercospora leaf spot disease (CLS) incidence for the TX2WSE diploid rose population evaluated in Texas in 2018 (A), 2020 (B), and 2021 (C) in Somerville (SV). [file Image_4.png]

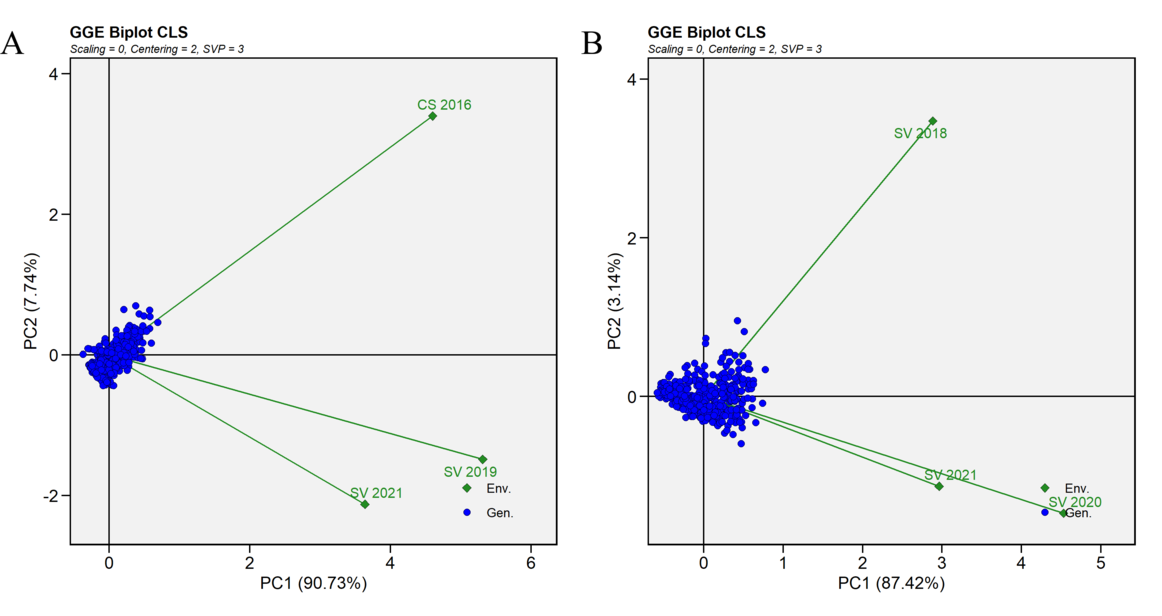

Supplement: Supplementary Figure 5 — Genotype plus genotype × environment (GGE) biplot representing the discrimination and representativeness of environments for the cercospora leaf spot disease (CLS) incidence in Texas College Station (CS) in 2016, Somerville (SV) in 2019 and 2021 for TX2WOB population (A), and SV in 2018, 2020, and 2021 for TX2WSE population (B). [file Image_5.png]

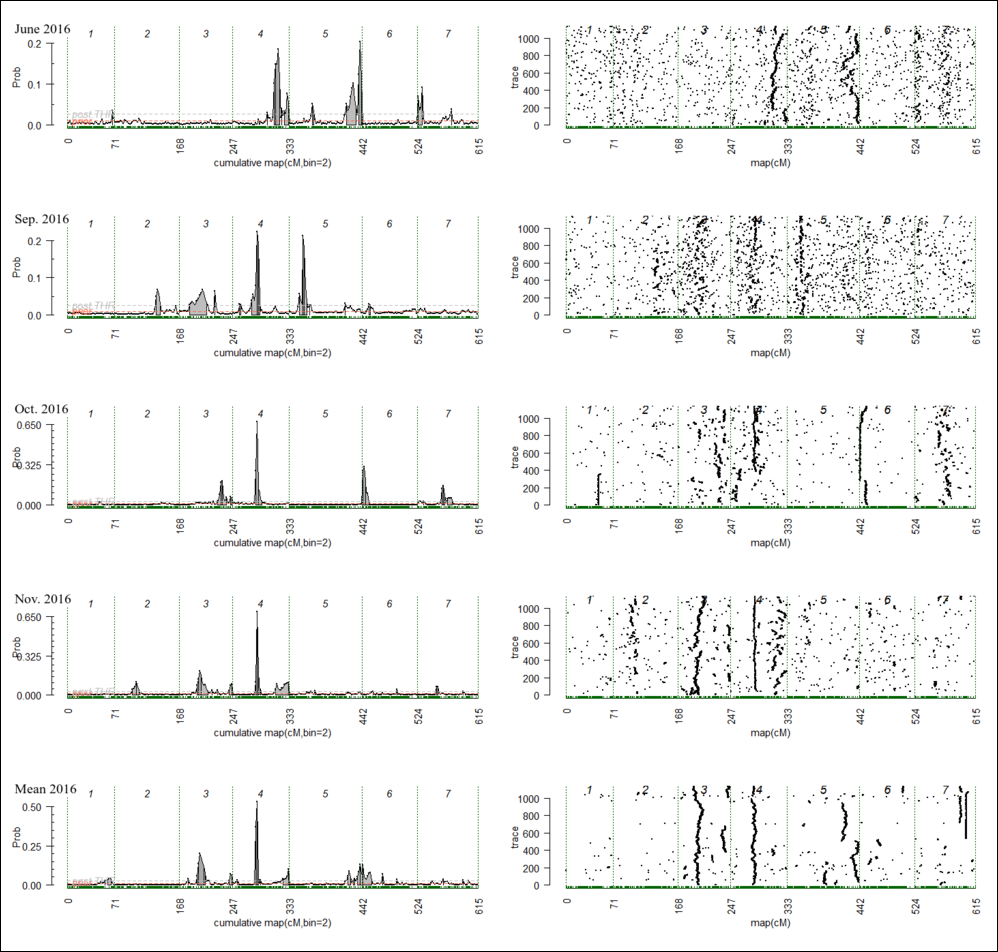

Supplement: Supplementary Figure 6 — Posterior positions (left) and trace samples QTL positions (right) based on an additive model performed using Visual FlexQTL software for cercospora leaf spot incidence in June, Sep., Oct., Nov., and the overall mean in 2016 for 11 diploid rose populations (TX2WOB) in College Station, Texas. [file Image_6.png]

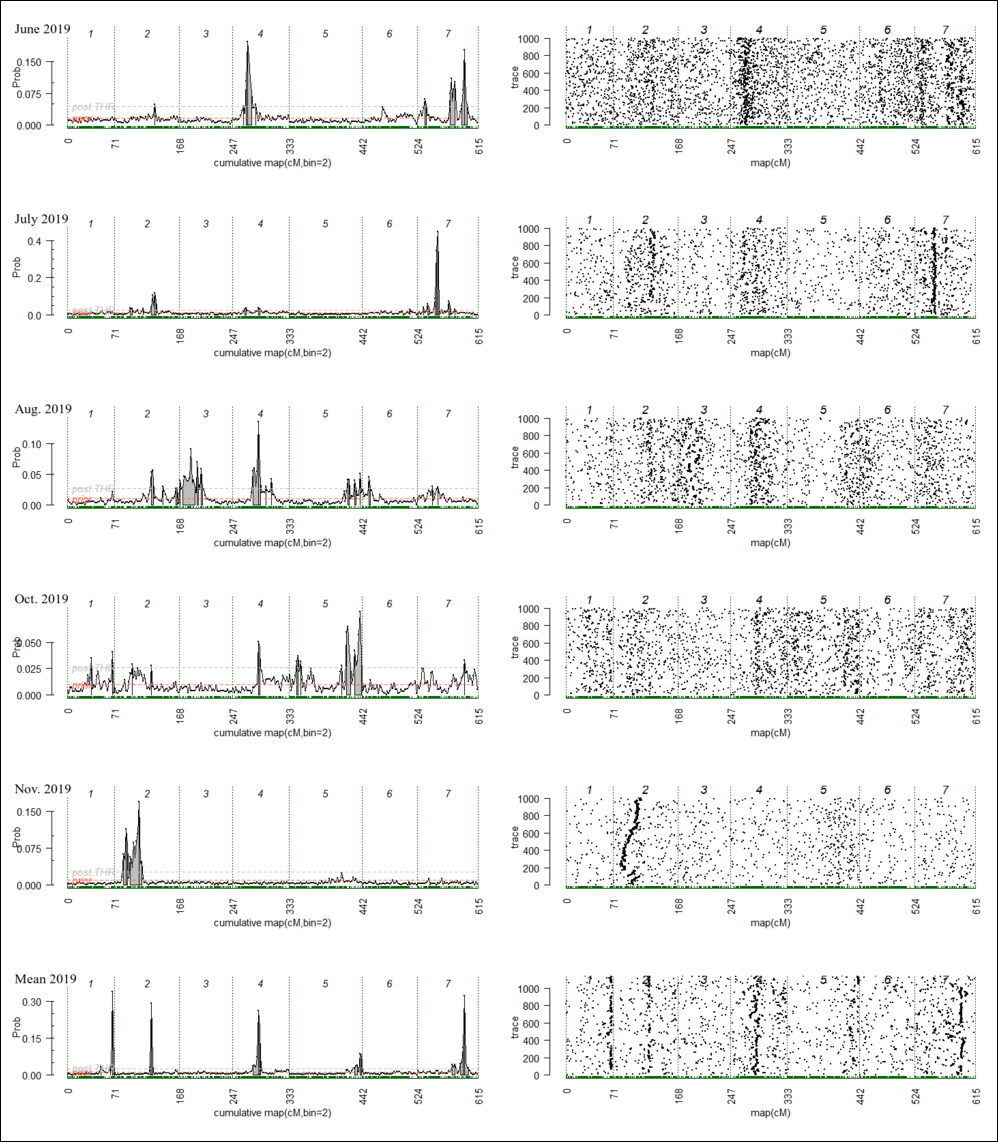

Supplement: Supplementary Figure 7 — Posterior positions (left) and trace samples QTL positions (right) based on an additive model performed using Visual FlexQTL software for cercospora leaf spot incidence in June, July, Aug., Oct., Nov., and the overall mean in 2019 for 10 diploid rose populations (TX2WOB) in Somerville, Texas. [file Image_7.png]

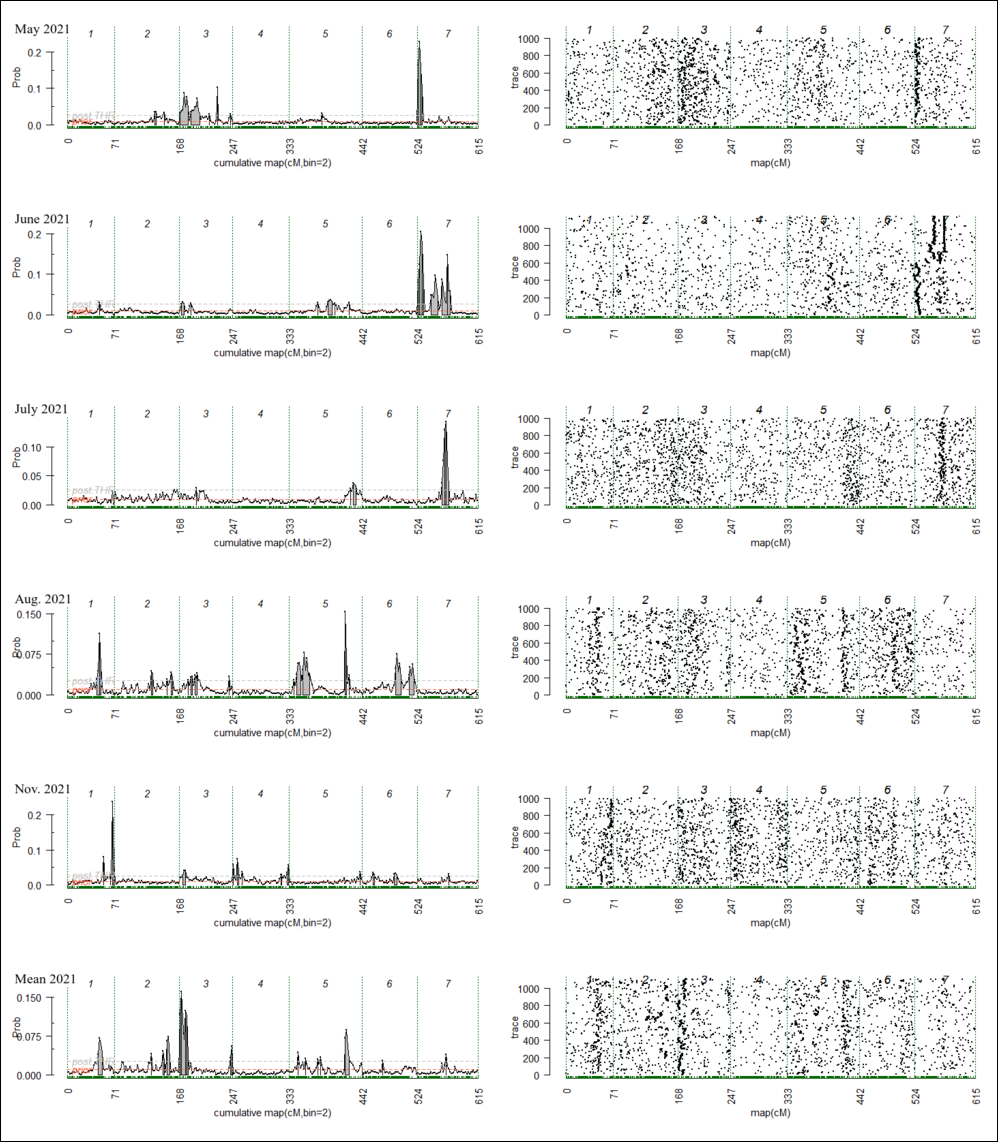

Supplement: Supplementary Figure 8 — Posterior positions (left) and trace samples QTL positions (right) based on an additive model performed using Visual FlexQTL software for cercospora leaf spot incidence in May, June, July, Aug., Nov., and the overall mean in 2021 for 10 diploid rose populations (TX2WOB) in Somerville, Texas. [file Image_8.png]

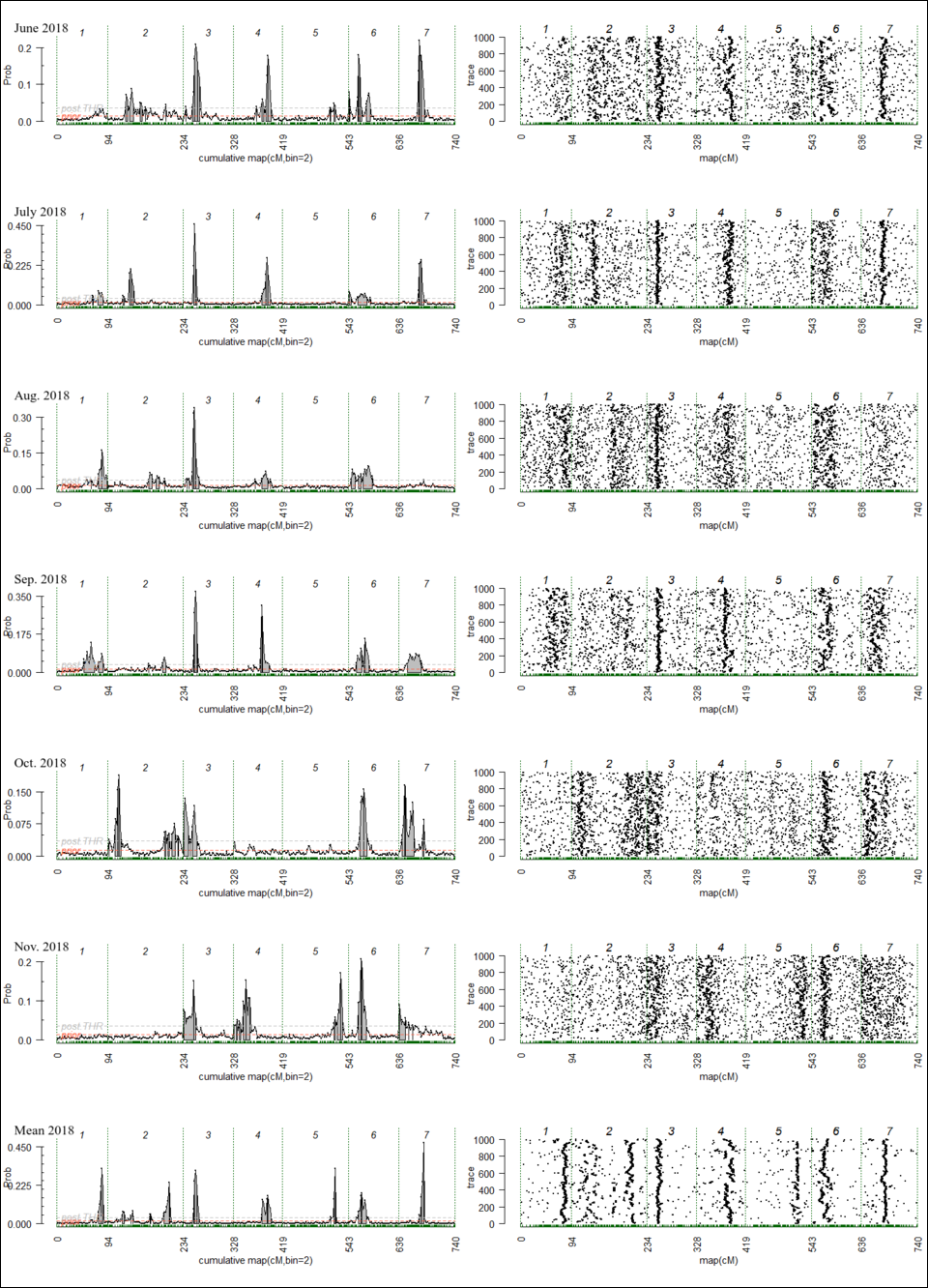

Supplement: Supplementary Figure 9 — Posterior positions (left) and trace samples QTL positions (right) based on an additive model performed using Visual FlexQTL software for cercospora leaf spot incidence in June, July, Aug., Sep., Oct., Nov., and the overall mean in 2018 for six diploid rose populations (TX2WSE) in Somerville, Texas [file Image_9.png]

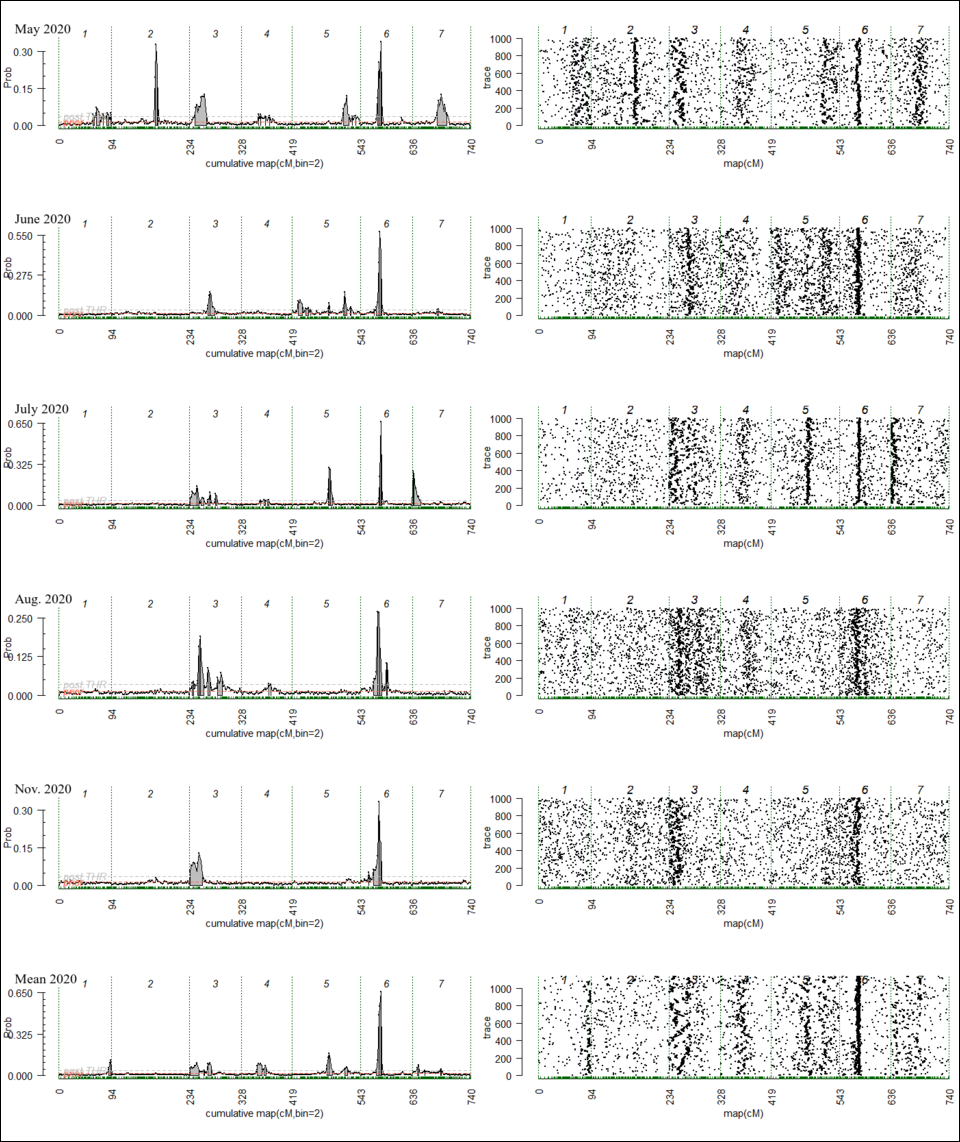

Supplement: Supplementary Figure 10 — Posterior positions (left) and trace samples QTL positions (right) based on an additive model performed using Visual FlexQTL software for cercospora leaf spot incidence in May, June, July, Aug., Nov., and the overall mean in 2020 for six diploid rose populations (TX2WSE) in Somerville, Texas. [file Image_10.png]

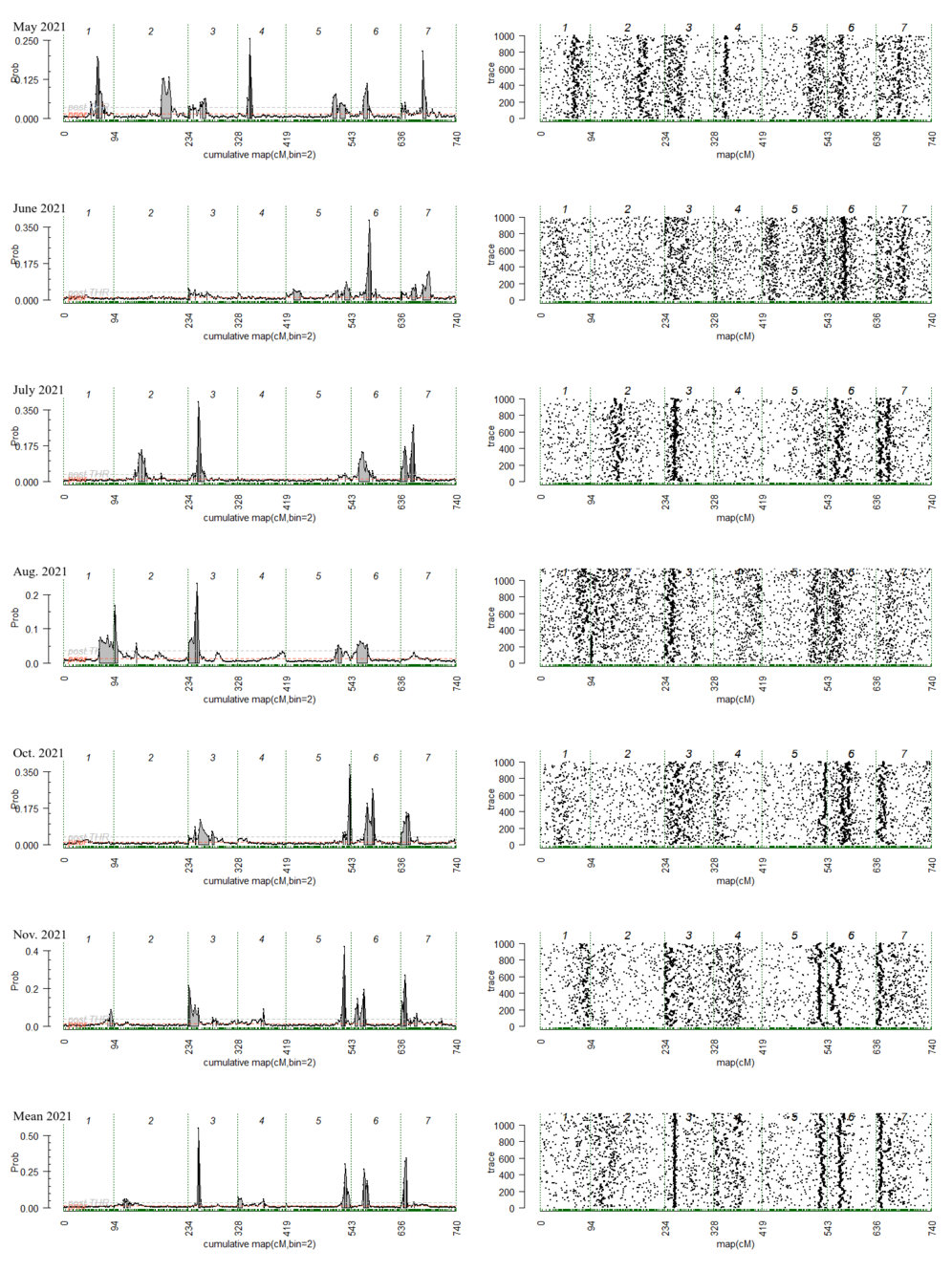

Supplement: Supplementary Figure 11 — Posterior positions (left) and trace samples QTL positions (right) based on an additive model performed using Visual FlexQTL software for cercospora leaf spot incidence in May, June, July, Aug., Oct., Nov., and the overall mean in 2021 for six diploid rose populations (TX2WSE) in Somerville, Texas. [file Image_11.png]

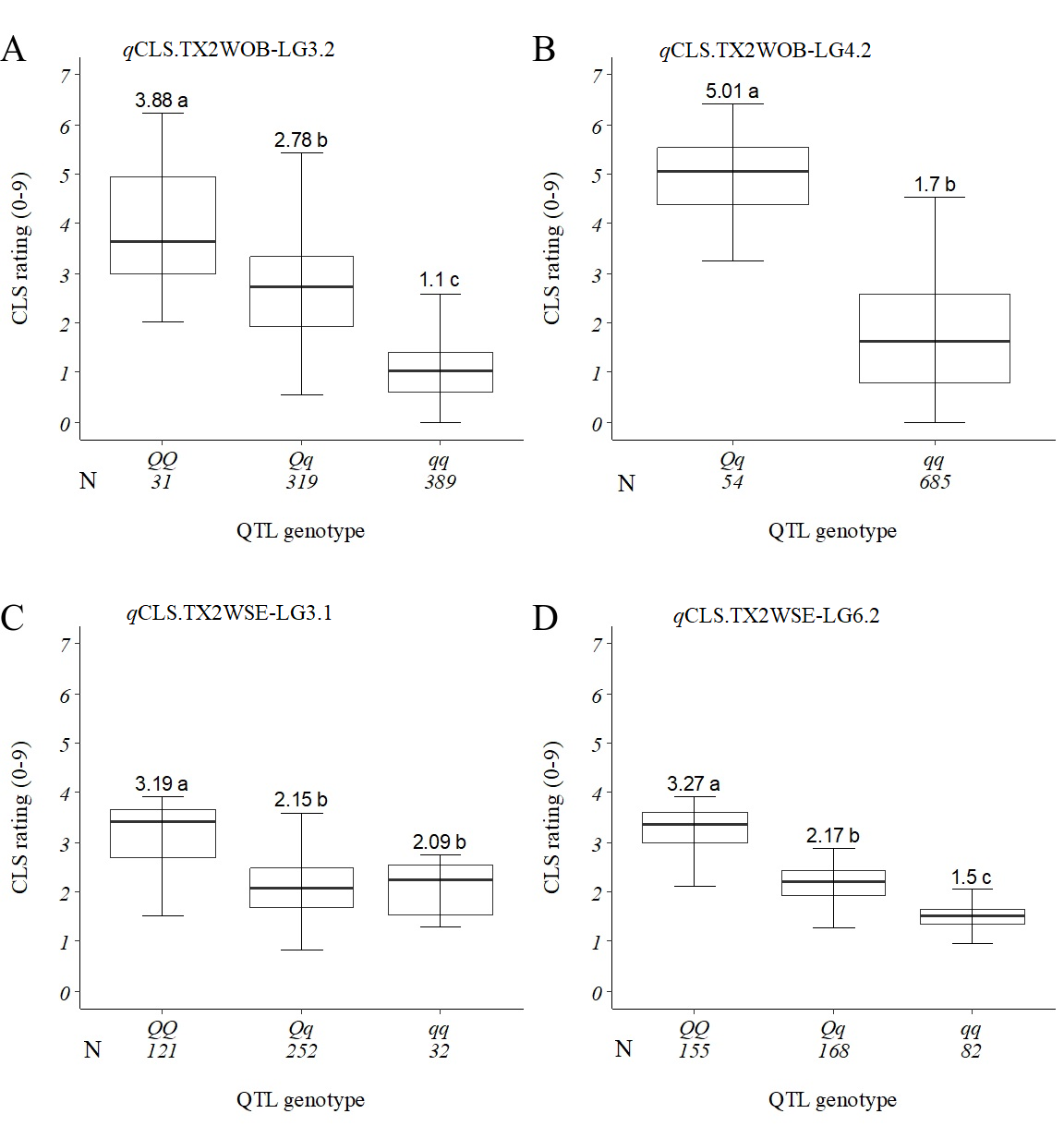

Supplement: Supplementary Figure 12 — Probable QTL genotype at the signal peak from all progenies for cercospora leaf spot QTLs of diploid rose populations qCLS.TX2WOB-LG3.2 (A) and qCLS.TX2WOB-LG4.2 (B) for TX2WOB and qCLS.TX2WSE-LG3.1 (C) and qCLS.TX2WSE-LG6.2 (D) for TX2WSE. [file Image_12.png]

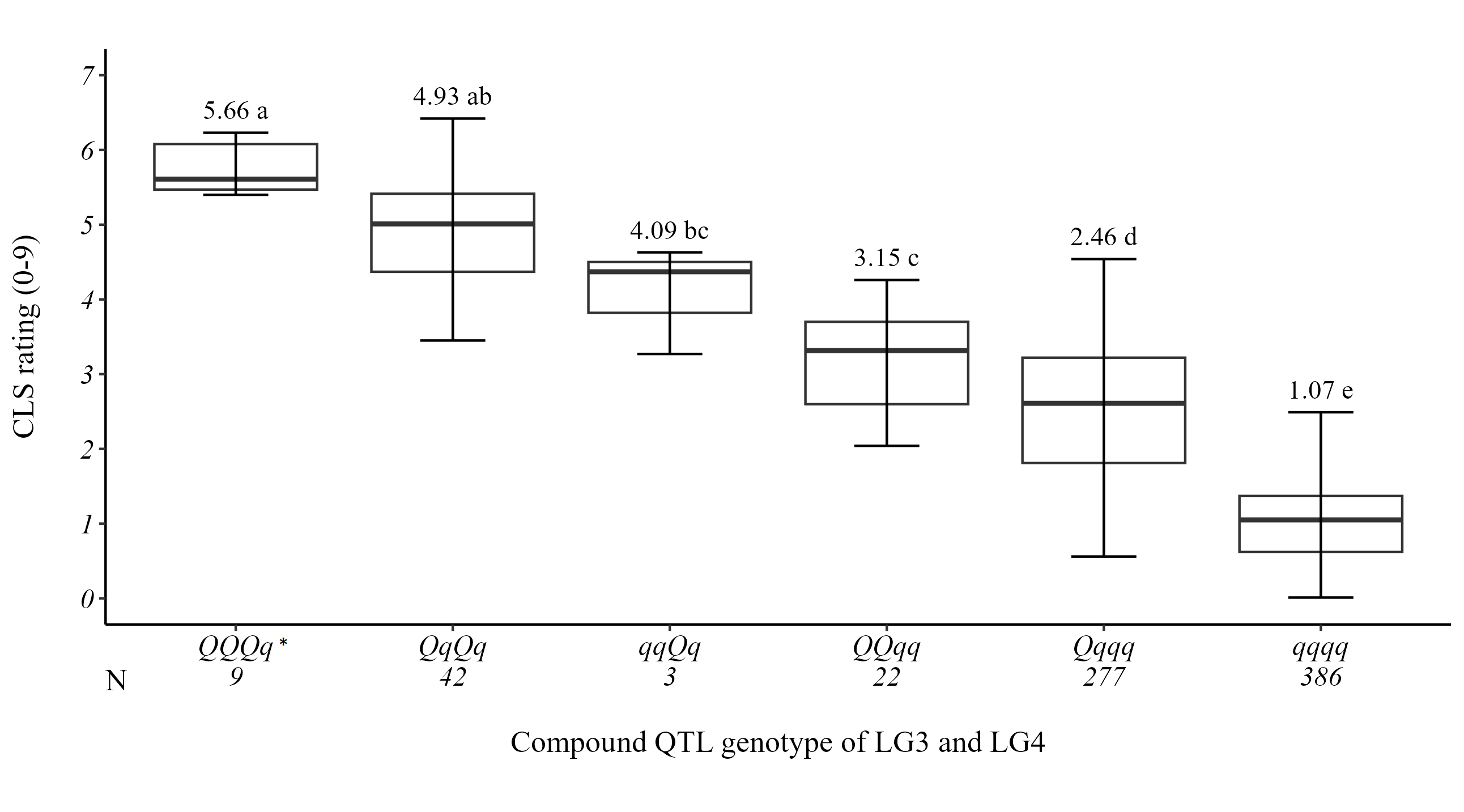

Supplement: Supplementary Figure 13 — Analysis of the compound QTL genotypes from qCLS.TX2WOB-LG3.2 and qCLS.TX2WOB-LG4.2 on cercospora leaf spot disease rating from all progenies in 11 diploid rose populations (TX2WOB). [file Image_13.png]

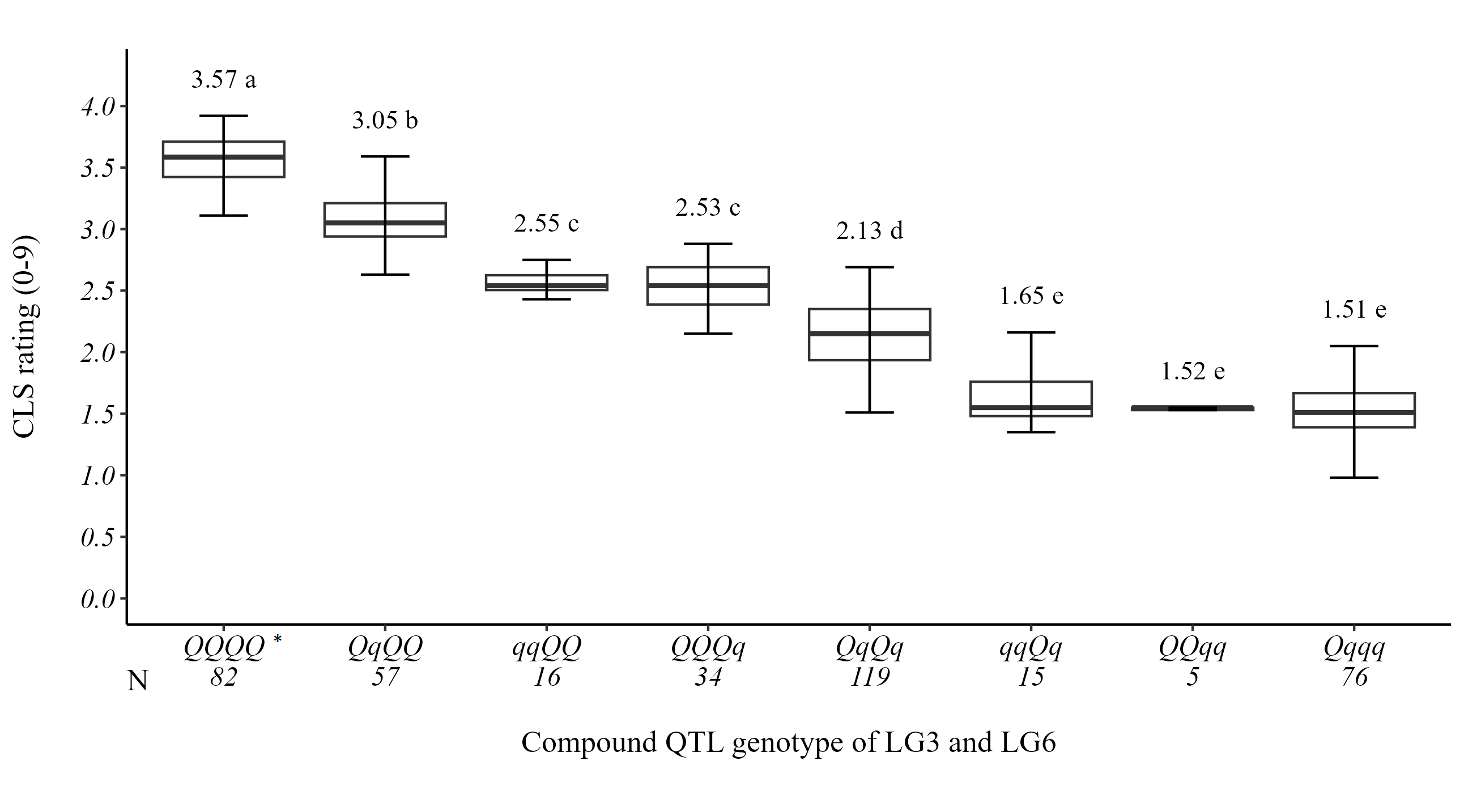

Supplement: Supplementary Figure 14 — Analysis of the compound QTL genotypes from qCLS.TX2WSE-LG3.1 and qCLS.TX2WSE-LG6.2 on cercospora leaf spot disease rating from all progenies in six diploid rose populations (TX2WSE). [file Image_14.png]
