# Supplementary material for: Pedigree-based analysis in multi-parental diploid rose populations reveals QTLs for cercospora leaf spot disease resistance
Source: Front Plant Sci. 2023 Jan 6;13:1082461. doi: 10.3389/fpls.2022.1082461 (PMC9859674; doi:10.3389/fpls.2022.1082461)
Supplement: Supplementary file 15 [file Table_1.docx]

Supplementary tables

| **Supplementary Table 1**. Parentage and number of progenies for 11 diploid rose populations that constitute the multi-parental TX2WOB population used for QTL analysis for cercospora leaf spot disease resistance in College Station, Texas in 2016 and Somerville, Texas in 2019 and 2021. | | | | | |
| --- | --- | --- | --- | --- | --- |
| Family | Female Parent | Male Parent | 2016 | 2019 | 2021 |
| J14-3×LC | J06-20-14-3 | Little Chief | 57 | 20 | 31 |
| J14-3×VS | J06-20-14-3 | Vineyard Song | 75 | 43 | 49 |
| J4-6×RF | J06-28-4-6 | Red Fairy | 57 | - | 1 |
| OB×J3-6 | Old Blush | J06-30-3-6 | 74 | 40 | 43 |
| OB×RF | Old Blush | Red Fairy | 61 | 32 | 43 |
| J14-3×RF | J06-20-14-3 | Red Fairy | 120 | 5 | 34 |
| J14-3×SC | J06-20-14-3 | Sweet Chariot | 52 | 34 | 40 |
| M4-4×SC | M4-4 | Sweet Chariot | 6 | 2 | 6 |
| OB×M4-4 | Old Blush | M4-4 | 9 | 8 | 8 |
| SC×J14-3 | Sweet Chariot | J06-20-14-3 | 21 | 16 | 21 |
| SC×M4-4 | Sweet Chariot | M4-4 | 49 | 18 | 22 |
| Total |  |  | 581 | 218 | 298 |

| **Supplementary Table 2**. Parentage and number of progenies for six diploid rose populations that constitute the TX2WSE multi-parental population used for QTL analysis for cercospora leaf spot disease resistance in 2018, 2020, and 2021 Somerville, Texas. | | | | | |
| --- | --- | --- | --- | --- | --- |
| Family | Female Parent | Male Parent | 2018 | 2020 | 2021 |
| J14-3×PH | J06-20-14-3 | Papa Hemeray | 135 | 128 | 123 |
| M4-4×SE | M4-4 | Srdce Europy | 32 | 32 | 32 |
| PH×SEB-ARE | Papa Hemeray | *R. palustris* f. *plena* EB-ARE | 10 | 10 | 10 |
| SET-ARE×OL | *R. setigera*-ARE | Ole | 24 | 22 | 24 |
| T7-20×SE | TAMU7-20 | Srdce Europy | 94 | 93 | 92 |
| T7-30×SE | TAMU7-30 | Srdce Europy | 82 | 81 | 81 |
| Total |  |  | 377 | 366 | 362 |

| **Supplementary Table 3**. Statistical summary of the five individual diploid rose maps and the integrated consensus map (TX2WOB ICM) by linkage group (LG). | | | | | | | |
| --- | --- | --- | --- | --- | --- | --- | --- |
|  | | | | | | Unique positions | |
| Family | LG | SNP | Length (cM) | Max gap (cM) | Density | SNP | Density |
| J14-3×LC | 1 | 532 | 70.6 | 7.1 | 7.5 | 177 | 2.5 |
| N=73^*^ | 2 | 405 | 96.3 | 5.4 | 4.2 | 151 | 1.6 |
|  | 3 | 342 | 75.8 | 10.3 | 4.5 | 125 | 1.6 |
|  | 4 | 247 | 73.8 | 8.2 | 3.3 | 131 | 1.8 |
|  | 5 | 189 | 107.1 | 11.3 | 1.8 | 110 | 1.0 |
|  | 6 | 386 | 82.3 | 11.2 | 4.7 | 178 | 2.2 |
|  | 7 | 256 | 90.7 | 5.1 | 2.8 | 101 | 1.1 |
| Total | | 2,357 | 596.6 | 11.3 | 4.1 | 973 | 1.7 |
| J14-3×VS | 1 | 406 | 84.6 | 14.0 | 4.8 | 147 | 1.7 |
| N=89 | 2 | 362 | 97.1 | 7.9 | 3.7 | 144 | 1.5 |
|  | 3 | 218 | 79.6 | 14.6 | 2.7 | 81 | 1.0 |
|  | 4 | 329 | 71.9 | 8.5 | 4.6 | 153 | 2.1 |
|  | 5 | 192 | 99.5 | 11.1 | 1.9 | 118 | 1.2 |
|  | 6 | 279 | 71.1 | 4.2 | 3.9 | 143 | 2.0 |
|  | 7 | 221 | 79.1 | 6.5 | 2.8 | 101 | 1.3 |
| Total | | 2,007 | 582.9 | 14.6 | 3.5 | 887 | 1.5 |
| J4-6×RF | 1 | 295 | 70.6 | 7.7 | 4.2 | 120 | 1.7 |
| N=68 | 2 | 321 | 95.6 | 8.4 | 3.4 | 134 | 1.4 |
|  | 3 | 196 | 74.4 | 13.8 | 2.6 | 80 | 1.1 |
|  | 4 | 352 | 85.9 | 8.7 | 4.1 | 149 | 1.7 |
|  | 5 | 200 | 108.9 | 5.3 | 1.8 | 100 | 0.9 |
|  | 6 | 243 | 69.5 | 3.3 | 3.5 | 131 | 1.9 |
|  | 7 | 196 | 75.8 | 6.1 | 2.6 | 98 | 1.3 |
| Total | | 1,803 | 580.7 | 13.8 | 3.2 | 812 | 1.4 |
| OB×J3-6 | 1 | 515 | 87.7 | 17.1 | 5.9 | 183 | 2.1 |
| N=97 | 2 | 242 | 83.4 | 9.4 | 2.9 | 94 | 1.1 |
|  | 3 | 147 | 64.1 | 6.9 | 2.3 | 65 | 1.0 |
|  | 4 | 214 | 71.4 | 9.8 | 3.0 | 108 | 1.5 |
|  | 5 | 378 | 97.9 | 5.5 | 3.9 | 203 | 2.1 |
|  | 6 | 311 | 75.6 | 6.7 | 4.1 | 165 | 2.2 |
|  | 7 | 104 | 36.0 | 3.5 | 2.9 | 52 | 1.4 |
| Total | | 1,911 | 516.1 | 17.1 | 3.6 | 870 | 1.6 |
| OB×RF | 1 | 222 | 70.6 | 8.6 | 3.1 | 69 | 1.0 |
| N=88 | 2 | 401 | 94.6 | 11.9 | 4.2 | 154 | 1.6 |
|  | 3 | 114 | 74.6 | 13.2 | 1.5 | 56 | 0.8 |
|  | 4 | 316 | 85.9 | 4.3 | 3.7 | 203 | 2.4 |
|  | 5 | 443 | 97.9 | 5.5 | 4.5 | 225 | 2.3 |
|  | 6 | 56 | 103.2 | 28.5 | 0.5 | 52 | 0.5 |
|  | 7 | 137 | 89.4 | 30.8 | 1.5 | 74 | 0.8 |
| Total | | 1,689 | 616.2 | 30.8 | 2.7 | 833 | 1.3 |
| Consensus | 1 | 757 | 87.7 | 14.0 | 8.6 | 252 | 2.9 |
| N=415 | 2 | 779 | 97.1 | 3.8 | 8.0 | 308 | 3.2 |
|  | 3 | 518 | 79.6 | 3.5 | 6.5 | 198 | 2.5 |
|  | 4 | 662 | 85.9 | 4.0 | 7.7 | 319 | 3.7 |
|  | 5 | 625 | 108.9 | 4.8 | 5.7 | 318 | 2.9 |
|  | 6 | 619 | 103.2 | 9.4 | 6.0 | 288 | 2.8 |
|  | 7 | 507 | 90.7 | 4.9 | 5.6 | 213 | 2.3 |
| Total | | 4,467 | 653.1 | 14.0 | 6.9 | 1,896 | 2.9 |
| * Number of individuals | | | | | | | |

| **Supplementary Table 4**. Statistical summary of the three individual diploid rose maps and the integrated consensus map (TX2WSE ICM) by linkage group (LG). | | | | | | | |
| --- | --- | --- | --- | --- | --- | --- | --- |
|  | | | | | | Unique positions | |
| Family | LG | SNP | Length (cM) | Max gap (cM) | Density | SNP | Density |
| J14-3×PH | 1 | 949 | 95.7 | 3.2 | 9.9 | 876 | 9.2 |
| N=138^*^ | 2 | 1,179 | 146.1 | 2.6 | 8.1 | 1,076 | 7.4 |
|  | 3 | 1,093 | 94.1 | 4.2 | 11.6 | 976 | 10.4 |
|  | 4 | 1,017 | 93.6 | 1.8 | 10.9 | 919 | 9.8 |
|  | 5 | 1,474 | 130.6 | 2.9 | 11.3 | 1,356 | 10.4 |
|  | 6 | 1,713 | 89.0 | 3.2 | 19.2 | 1,358 | 15.3 |
|  | 7 | 871 | 110.4 | 1.9 | 7.9 | 801 | 7.3 |
| Total | | 8,296 | 759.5 | 4.2 | 10.9 | 7,362 | 9.7 |
| T7-20×SE | 1 | 390 | 75.8 | 1.6 | 5.1 | 359 | 4.7 |
| N=94 | 2 | 677 | 97.6 | 5.5 | 6.9 | 561 | 5.7 |
|  | 3 | 688 | 87.2 | 2.5 | 7.9 | 604 | 6.9 |
|  | 4 | 506 | 78.2 | 2.2 | 6.5 | 458 | 5.9 |
|  | 5 | 816 | 107.6 | 2.2 | 7.6 | 757 | 7.0 |
|  | 6 | 1,125 | 94.5 | 3.0 | 11.9 | 887 | 9.4 |
|  | 7 | 816 | 87.3 | 3.2 | 9.3 | 733 | 8.4 |
| Total | | 5,018 | 628.2 | 5.5 | 8.0 | 4,359 | 6.9 |
| T7-30×SE | 1 | 677 | 97.0 | 1.9 | 7.0 | 619 | 6.4 |
| N=82 | 2 | 1,149 | 146.5 | 5.7 | 7.8 | 999 | 6.8 |
|  | 3 | 730 | 95.5 | 3.1 | 7.6 | 660 | 6.9 |
|  | 4 | 565 | 88.5 | 5.9 | 6.4 | 510 | 5.8 |
|  | 5 | 748 | 104.7 | 2.8 | 7.1 | 694 | 6.6 |
|  | 6 | 1,141 | 103.8 | 2.3 | 11.0 | 953 | 9.2 |
|  | 7 | 954 | 119.9 | 2.8 | 8.0 | 877 | 7.3 |
| Total | | 5,964 | 755.8 | 5.9 | 7.9 | 5,312 | 7.0 |
| Consensus | 1 | 346 | 97.7 | 3.4 | 3.5 | 168 | 1.7 |
| N=314 | 2 | 467 | 146.1 | 4.5 | 3.2 | 197 | 1.3 |
|  | 3 | 345 | 93.9 | 3.4 | 3.7 | 141 | 1.5 |
|  | 4 | 321 | 93.6 | 4.5 | 3.4 | 139 | 1.5 |
|  | 5 | 417 | 123.6 | 5.9 | 3.4 | 194 | 1.6 |
|  | 6 | 389 | 92.8 | 4.2 | 4.2 | 178 | 1.9 |
|  | 7 | 392 | 110.6 | 6.8 | 3.5 | 145 | 1.3 |
| Total | | 2,677 | 758.2 | 6.8 | 3.5 | 1,162 | 1.5 |
| * Number of individuals | | | | | | | |

| **Supplementary Table 5**. Descriptive statistics of cercospora leaf spot (CLS) disease incidence evaluated in Texas on 11 diploid rose populations (TX2WOB multi-parental population) in 2016 in College Station (CS) and a subset of ten populations in Somerville (SV) in 2019 and 2021. | | | | | | | |
| --- | --- | --- | --- | --- | --- | --- | --- |
| Trait (0-9 scale) | | N | Mean | $\sigma_{p}^{2}$ ^y^ | SD | Min | Max |
| CS 2016 | June | 428 | 2.5 | 5.25 | 2.11 | 0.0 | 7.0 |
|  | Sep. | 476 | 0.9 | 1.48 | 1.21 | 0.0 | 4.0 |
|  | Oct. | 529 | 1.7 | 3.67 | 1.92 | 0.0 | 7.0 |
|  | Nov. | 542 | 2.3 | 4.41 | 1.75 | 0.0 | 6.5 |
|  | Mean | 575 | 2.7 | 2.50 | 1.89 | 0.0 | 8.0 |
| SV 2019 | June | 206 | 2.2 | 1.25 | 1.12 | 0.5 | 5.5 |
|  | July | 225 | 3.5 | 3.23 | 1.80 | 0.5 | 8.0 |
|  | Aug. | 222 | 2.9 | 2.04 | 1.43 | 0.5 | 7.5 |
|  | Oct. | 215 | 3.1 | 1.17 | 1.08 | 0.5 | 6.0 |
|  | Nov. | 220 | 3.7 | 1.66 | 1.29 | 1.0 | 6.6 |
|  | Mean | 227 | 3.0 | 0.95 | 0.98 | 0.8 | 5.3 |
| SV 2021 | May | 294 | 2.6 | 0.80 | 0.89 | 0.0 | 5.0 |
|  | June | 289 | 2.0 | 0.45 | 0.67 | 0.0 | 4.0 |
|  | July | 291 | 2.3 | 0.66 | 0.81 | 0.0 | 5.0 |
|  | Aug. | 285 | 1.8 | 0.67 | 0.82 | 0.0 | 4.0 |
|  | Nov. | 294 | 2.8 | 0.80 | 0.89 | 0.0 | 6.0 |
|  | Mean | 298 | 2.3 | 0.26 | 0.50 | 0.4 | 3.6 |

| **Supplementary Table 6**. Monthly maximum (Max), minimum (Min), and average temperature, relative humidity, and precipitation in College Station (CS) in 2016, Somerville (SV) in 2018, 2019, 2020, and 2021 in Texas. | | | | | | | |
| --- | --- | --- | --- | --- | --- | --- | --- |
| Location | Year | Month | Temperature (°C) | | | Humidity | Precipitation |
|  |  |  | Max | Min | Average | (%) | (mm) |
| CS | 2016 | Jan. | 16.1 | 4.9 | 10.3 | 69.0 | 33.3 |
|  |  | Feb. | 20.7 | 8.2 | 14.2 | 62.8 | 33.5 |
|  |  | Mar. | 23.9 | 13.1 | 18.0 | 72.9 | 112.5 |
|  |  | Apr. | 25.8 | 15.3 | 20.1 | 75.5 | 135.1 |
|  |  | May | 27.8 | 18.7 | 22.7 | 79.1 | 272.8 |
|  |  | June | 32.8 | 23.3 | 27.3 | 76.1 | 55.9 |
|  |  | July | 35.4 | 25.0 | 29.4 | 71.9 | 6.1 |
|  |  | Aug. | 33.6 | 23.9 | 28.0 | 78.0 | 226.6 |
|  |  | Sep. | 32.4 | 22.8 | 27.0 | 75.1 | 50.8 |
|  |  | Oct. | 29.6 | 17.8 | 22.9 | 73.2 | 54.6 |
|  |  | Nov. | 23.8 | 13.1 | 18.1 | 76.2 | 69.3 |
|  |  | Dec. | 17.6 | 8.9 | 13.2 | 72.5 | 70.4 |
|  |  | Total | 26.6 | 16.3 | 20.9 | 73.6 | 1120.9 |
| SV | 2018 | Jan. | 15.0 | 3.1 | 8.7 | 63.4 | 26.9 |
|  |  | Feb. | 17.5 | 9.2 | 13.0 | 87.0 | 47.2 |
|  |  | Mar. | 24.0 | 12.9 | 18.3 | 68.0 | 156.5 |
|  |  | Apr. | 24.5 | 12.3 | 18.4 | 69.3 | 37.6 |
|  |  | May | 31.4 | 20.6 | 25.4 | 74.7 | 52.8 |
|  |  | June | 33.9 | 24.1 | 28.0 | 77.2 | 47.8 |
|  |  | July | 35.4 | 24.1 | 29.0 | 70.4 | 37.8 |
|  |  | Aug. | 36.0 | 24.0 | 29.5 | 67.6 | 8.1 |
|  |  | Sep. | 30.6 | 19.9 | 24.2 | 81.1 | 175.5 |
|  |  | Oct. | 25.5 | 15.5 | 20.0 | 86.5 | 291.1 |
|  |  | Nov. | 18.2 | 8.2 | 13.0 | 78.9 | 141.5 |
|  |  | Dec. | 16.2 | 7.1 | 11.5 | 75.7 | 243.8 |
|  |  | Total | 25.7 | 15.1 | 20.0 | 74.9 | 1266.7 |
| SV | 2019 | Jan. | 15.4 | 5.2 | 10.1 | 76.6 | 122.2 |
|  |  | Feb. | 17.0 | 9.0 | 12.8 | 82.6 | 53.8 |
|  |  | Mar. | 20.4 | 10.5 | 15.2 | 71.0 | 31.8 |
|  |  | Apr. | 25.7 | 14.5 | 19.7 | 75.6 | 141.0 |
|  |  | May | 29.3 | 20.6 | 24.3 | 79.8 | 148.1 |
|  |  | June | 32.2 | 22.7 | 26.9 | 75.5 | 102.4 |
|  |  | July | 34.5 | 24.2 | 28.6 | 70.9 | 1.0 |
|  |  | Aug. | 36.4 | 25.6 | 29.9 | 70.3 | 42.7 |
|  |  | Sep. | 34.4 | 23.9 | 28.4 | 70.4 | 56.1 |
|  |  | Oct. | 27.8 | 16.1 | 21.3 | 67.7 | 77.7 |
|  |  | Nov. | 21.1 | 8.6 | 14.7 | 70.6 | 32.3 |
|  |  | Dec. | 19.9 | 6.8 | 12.8 | 66.5 | 14.2 |
|  |  | Total | 26.2 | 15.7 | 20.4 | 73.0 | 823.2 |
| SV | 2020 | Jan. | 19.1 | 8.7 | 13.9 | 71.9 | 63.2 |
|  |  | Feb. | 21.0 | 5.4 | 13.6 | 68.0 | 567.9 |
|  |  | Mar. | 25.4 | 16.2 | 20.2 | 77.7 | 63.5 |
|  |  | Apr. | 25.8 | 14.8 | 20.1 | 74.4 | 98.0 |
|  |  | May | 30.0 | 19.3 | 24.0 | 71.5 | 69.9 |
|  |  | June | 32.6 | 22.9 | 27.4 | 69.2 | 40.6 |
|  |  | July | 35.3 | 25.1 | 28.9 | 73.6 | 66.3 |
|  |  | Aug. | 36.2 | 24.2 | 29.6 | 64.9 | 18.0 |
|  |  | Sep. | 30.0 | 21.0 | 24.8 | 76.1 | 95.8 |
|  |  | Oct. | 27.4 | 15.6 | 20.9 | 70.4 | 9.4 |
|  |  | Nov. | 24.7 | 12.5 | 18.3 | 67.1 | 24.4 |
|  |  | Dec. | 18.4 | 5.7 | 11.8 | 69.2 | 91.9 |
|  |  | Total | 27.2 | 16.0 | 21.2 | 71.2 | 1209.0 |
| SV | 2021 | Jan. | 17.1 | 5.9 | 11.3 | 67.7 | 94.5 |
|  |  | Feb. | 14.1 | 3.7 | 8.7 | 72.6 | 28.2 |
|  |  | Mar. | 23.3 | 11.6 | 17.1 | 66.6 | 43.9 |
|  |  | Apr. | 24.9 | 14.8 | 19.5 | 69.1 | 77.7 |
|  |  | May | 28.3 | 19.9 | 23.5 | 76.5 | 160.3 |
|  |  | June | 32.6 | 23.8 | 27.6 | 73.9 | 73.9 |
|  |  | July | 32.8 | 24.0 | 27.6 | 76.3 | 101.9 |
|  |  | Aug. | 34.3 | 24.8 | 29.0 | 70.8 | 55.6 |
|  |  | Sep. | 33.3 | 21.5 | 26.7 | 62.8 | 6.1 |
|  |  | Oct. | 29.2 | 18.0 | 23.0 | 66.3 | 107.2 |
|  |  | Nov. | 22.3 | 10.2 | 15.9 | 68.5 | 44.2 |
|  |  | Dec. | 24.1 | 14.1 | 18.5 | 77.3 | 36.1 |
|  |  | Total | 26.4 | 16.1 | 20.8 | 70.7 | 829.6 |

| **Supplementary Table 7**. Descriptive statistics of cercospora leaf spot (CLS) incidence evaluated in Texas on six diploid rose populations (TX2WSE multi-parental population) in 2018, 2020, and 2021 Somerville (SV). | | | | | | | |
| --- | --- | --- | --- | --- | --- | --- | --- |
| Loc. Year | Month | N | Mean | $\sigma_{p}^{2}$ ^y^ | SD | Min | Max |
| SV 2018 | June | 379 | 1.6 | 1.44 | 1.20 | 0.0 | 6.9 |
|  | July | 379 | 1.5 | 2.16 | 1.47 | 0.0 | 8.6 |
|  | Aug. | 379 | 1.4 | 2.43 | 1.56 | 0.0 | 8.3 |
|  | Sep. | 380 | 1.9 | 3.21 | 1.79 | 0.0 | 7.9 |
|  | Oct. | 378 | 2.0 | 2.11 | 1.45 | 0.0 | 7.8 |
|  | Nov. | 380 | 1.3 | 2.16 | 1.47 | 0.0 | 8.5 |
|  | Mean | 380 | 1.4 | 0.85 | 0.92 | 0.1 | 5.0 |
| SV 2020 | May | 365 | 3.7 | 5.65 | 2.38 | 0.0 | 8.8 |
|  | June | 365 | 3.4 | 5.56 | 2.36 | 0.0 | 8.0 |
|  | July | 369 | 4.9 | 5.06 | 2.25 | 0.0 | 9.0 |
|  | Aug. | 367 | 2.5 | 2.81 | 1.67 | 0.0 | 7.3 |
|  | Nov. | 368 | 1.5 | 2.24 | 1.50 | 0.0 | 7.0 |
|  | Mean | 369 | 3.2 | 2.19 | 1.48 | 0.0 | 6.4 |
| SV 2021 | May | 365 | 0.6 | 0.76 | 0.87 | 0.0 | 5.5 |
|  | June | 365 | 2.8 | 6.17 | 2.48 | 0.0 | 9.0 |
|  | July | 365 | 0.9 | 1.62 | 1.27 | 0.0 | 6.0 |
|  | Aug. | 365 | 1.0 | 1.77 | 1.33 | 0.0 | 6.0 |
|  | Oct. | 365 | 1.0 | 1.42 | 1.19 | 0.0 | 6.0 |
|  | Nov. | 365 | 2.8 | 4.56 | 2.13 | 0.0 | 8.0 |
|  | Mean | 365 | 1.5 | 1.45 | 1.20 | 0.0 | 5.4 |

| **Supplementary Table 8**. Genetic variance ${(\sigma}_{g}^{2})$, genotype by environment variance ${(\sigma}_{g\times e}^{2})$, the genotype by environment variance relative to the genetic variance $({\sigma_{g\times e}^{2}}/{\sigma_{g}^{2})}$, and broad-sense heritability (H^2^) assessed in Texas for cercospora leaf spot (CLS) in 11 diploid rose populations (TX2WOB) in College Station (CS) 2016, and a subset of ten populations in Somerville (SV) 2019 and 2021, and on six diploid rose populations (TX2WSE) in SV 2018, 2020, and 2021. | | | | |
| --- | --- | --- | --- | --- |
| Population / Environment | $\sigma_{g}^{2}$ | $\sigma_{g\times e}^{2}$ | ${\sigma_{g\times e}^{2}}/{\sigma_{g}^{2}}$ | H^2^ |
| TX2WOB / CS 2016, SV 2019 & SV 2021 | 0.56 | 1.19 | 2.13 | 0.58 |
| TX2WSE / SV 2018, SV 2020 & SV 2021 | 1.00 | 1.33 | 1.33 | 0.69 |

| **Supplementary Table 9**. Correlation coefficient (r) for cercospora leaf spot (CLS) incidence between environments evaluated in Texas on TX2WOB populations (11 diploid rose populations) in College Station (CS) 2016, Somerville (SV) 2019 and 2021, TX2WSE populations (six diploid rose populations) in SV 2018, 2020, and 2021. | | | |
| --- | --- | --- | --- |
| Population | CLS | CLS | r |
| TX2WOB | CS 2016 | SV 2019 | 0.85 |
|  | CS 2016 | SV 2021 | 0.77 |
|  | SV 2019 | SV 2021 | 0.94 |
| TX2WSE | SV 2018 | SV 2020 | 0.62 |
|  | SV 2018 | SV 2021 | 0.55 |
|  | SV 2020 | SV 2021 | 0.71 |

| **Supplementary Table 10**. QTLs mapped for the cercospora leaf spot (CLS) evaluated in Texas on 11 diploid rose populations (TX2WOB) across multiple months in 2016 in College Station (CS) and on a ten-population subset in 2019 and 2021 in Somerville (SV). | | | | | | | | | | | |
| --- | --- | --- | --- | --- | --- | --- | --- | --- | --- | --- | --- |
|  | | | | | | | | | | *2ln(BF)* | |
| Loc. Year | Month | MCMC | Records | μ | σ^2^_p_ | σ^2^_e_ | σ^2^_A_ | h^2^ | LG | 1/0 | 2/1 |
| CS 2016 | June | 125,000 | 428 | 2.52 | 5.25 | 2.95 | 2.30 | 0.44 | 4 | 9.7 | -0.3 |
|  |  |  |  |  |  |  |  |  | 5 | 7.8 | 0.8 |
|  | Sep. | 200,000 | 476 | 0.90 | 1.48 | 1.11 | 0.36 | 0.25 | 3 | 3.4 | -0.3 |
|  |  |  |  |  |  |  |  |  | 4 | 28.9 | 1.4 |
|  |  |  |  |  |  |  |  |  | 5 | 4.5 | 0.4 |
|  | Oct. | 800,000 | 529 | 1.68 | 3.67 | 1.72 | 1.96 | 0.53 | 3 | 9.3 | -1.2 |
|  |  |  |  |  |  |  |  |  | 4 | 10.8 | 3.1 |
|  |  |  |  |  |  |  |  |  | 6 | 29.4 | -4.4 |
|  |  |  |  |  |  |  |  |  | 7 | 3.1 | 0.2 |
|  | Nov. | 250,000 | 539 | 2.33 | 4.41 | 2.09 | 2.32 | 0.53 | 3 | 28.8 | 1.3 |
|  |  |  |  |  |  |  |  |  | 4 | 25.8 | 6.2 |
|  | Mean | 200,000 | 575 | 1.70 | 2.50 | 0.93 | 1.57 | 0.63 | 3 | 28.9 | 1.3 |
|  |  |  |  |  |  |  |  |  | 4 | 12.6 | 2.7 |
|  |  |  |  |  |  |  |  |  | 5 | 2.9 | -3.2 |
|  |  |  |  |  |  |  |  |  | 6 | 3.1 | -1.8 |
| SV 2019 | Jun. | 100,000 | 206 | 2.23 | 1.25 | 0.75 | 0.51 | 0.41 | 4 | 5.7 | 0.8 |
|  |  |  |  |  |  |  |  |  | 7 | 3.5 | 1.3 |
|  | Jul. | 100,000 | 225 | 3.46 | 3.23 | 1.40 | 1.83 | 0.57 | 2 | 3.0 | 0.1 |
|  |  |  |  |  |  |  |  |  | 7 | 7.3 | 2.8 |
|  | Aug. | 100,000 | 222 | 2.85 | 2.04 | 1.24 | 0.81 | 0.40 | 3 | 3.8 | -0.2 |
|  |  |  |  |  |  |  |  |  | 4 | 3.2 | -0.6 |
|  | Oct. | 100,000 | 215 | 3.11 | 1.17 | 0.89 | 0.28 | 0.24 | 5 | 2.2 | 0.9 |
|  | Nov. | 100,000 | 220 | 3.74 | 1.66 | 1.17 | 0.49 | 0.29 | 2 | 12.1 | -0.2 |
|  | Mean | 200,000 | 227 | 2.98 | 0.95 | 0.37 | 0.59 | 0.62 | 1 | 2.1 | -0.2 |
|  |  |  |  |  |  |  |  |  | 4 | 4.3 | 0.7 |
|  |  |  |  |  |  |  |  |  | 7 | 3.8 | 0.9 |
| SV 2021 | May | 100,000 | 294 | 2.57 | 0.80 | 0.63 | 0.17 | 0.21 | 3 | 4.4 | 1.4 |
|  |  |  |  |  |  |  |  |  | 7 | 3.2 | 0.4 |
|  | Jun. | 900,000 | 289 | 1.97 | 0.45 | 0.28 | 0.17 | 0.37 | 7 | 8.5 | 3.2 |
|  | Jul. | 100,000 | 291 | 2.27 | 0.66 | 0.55 | 0.11 | 0.17 | 7 | 2.6 | 0.3 |
|  | Aug. | 100,000 | 285 | 1.82 | 0.67 | 0.51 | 0.16 | 0.24 | 5 | 3.6 | 1.1 |
|  |  |  |  |  |  |  |  |  | 6 | 3.1 | 0.5 |
|  | Nov. | 100,000 | 294 | 2.83 | 0.80 | 0.66 | 0.13 | 0.17 | 1 | 2.6 | -0.1 |
|  | Mean | 100,000 | 298 | 2.34 | 0.26 | 0.17 | 0.09 | 0.35 | 3 | 4.6 | 0.4 |
|  |  |  |  |  |  |  |  |  | 5 | 2.0 | 0.2 |
| Markov chain Monte Carlo (MCMC) run length, phenotypic mean (*μ*), phenotypic variance (*σ^2^_P_*), residual variance(*σ^2^_e_*), additive variance(*σ^2^_A_*), narrow-sense heritability (*h^2^*), the linkage groups (LG) that QTLs were mapped on.  *2ln(BF)*. Bayes Factor, a measure quantifies the support from the data for the number of QTL(s) in the model (QTL evidence), after pair-wise model comparison (1/0, 2/1, and 3/2) such as ‘one-QTL model’ vs. ‘zero-QTL model, etc. *2ln(BF)* <0 = no evidence; 0-2 = hardly any; 2-5 = positive; 5-10 = strong; >10 = decisive. Bayes Factor will not be available (na) if either model does not have enough samples in the Markov chain. | | | | | | | | | | | |

| **Supplementary Table 11**. QTLs mapped for the cercospora leaf spots (CLS) resistance evaluated in Texas on six diploid rose populations (TX2WSE) across multiple months and overall mean in 2018, 2020, and 2021 Somerville (SV). | | | | | | | | | | | |
| --- | --- | --- | --- | --- | --- | --- | --- | --- | --- | --- | --- |
|  | | | | | | | | | | ***2ln(BF)*** | |
| ***Loc. Year*** | ***Month*** | ***MCMC*** | ***Records*** | ***μ*** | ***σ^2^_p_*** | ***σ^2^_e_*** | ***σ^2^_A_*** | ***h^2^*** | ***LG*** | ***1/0*** | ***2/1*** |
| *SV 2018* | June | 100,000 | 379 | 1.58 | 1.44 | 0.90 | 0.54 | 0.38 | 2 | 3.7 | 1.1 |
|  |  |  |  |  |  |  |  |  | 3 | 11.5 | 1.2 |
|  |  |  |  |  |  |  |  |  | 4 | 5.4 | -1.1 |
|  |  |  |  |  |  |  |  |  | 6 | 3.7 | 1.1 |
|  |  |  |  |  |  |  |  |  | 7 | 11.5 | 1.2 |
|  | July | 100,000 | 379 | 1.48 | 2.16 | 1.27 | 0.89 | 0.41 | 2 | 5.5 | 0.5 |
|  |  |  |  |  |  |  |  |  | 3 | 27.9 | -0.1 |
|  |  |  |  |  |  |  |  |  | 4 | 27.9 | 0.5 |
|  |  |  |  |  |  |  |  |  | 7 | 10.3 | -0.3 |
|  | Aug. | 100,000 | 379 | 1.43 | 2.43 | 1.64 | 0.80 | 0.33 | 1 | 4.1 | 0.9 |
|  |  |  |  |  |  |  |  |  | 3 | 27.7 | 0.8 |
|  |  |  |  |  |  |  |  |  | 6 | 8.6 | 0.9 |
|  | Sep. | 100,000 | 380 | 1.85 | 3.21 | 1.90 | 1.31 | 0.41 | 1 | 4.3 | 1.0 |
|  |  |  |  |  |  |  |  |  | 3 | 12.8 | -0.2 |
|  |  |  |  |  |  |  |  |  | 4 | 12.8 | -0.1 |
|  |  |  |  |  |  |  |  |  | 6 | 9.4 | -1.1 |
|  |  |  |  |  |  |  |  |  | 7 | 5.4 | -0.6 |
|  | Oct. | 100,000 | 378 | 2.01 | 2.11 | 1.53 | 0.58 | 0.28 | 2 | 4.4 | 3.5 |
|  |  |  |  |  |  |  |  |  | 3 | 6.3 | -0.4 |
|  |  |  |  |  |  |  |  |  | 6 | 6.8 | -0.6 |
|  |  |  |  |  |  |  |  |  | 7 | 27.2 | 1.2 |
|  | Nov. | 100,000 | 380 | 1.34 | 2.16 | 1.61 | 0.55 | 0.26 | 3 | 5.0 | 0.0 |
|  |  |  |  |  |  |  |  |  | 4 | 8.6 | 0.3 |
|  |  |  |  |  |  |  |  |  | 5 | 2.5 | -0.2 |
|  |  |  |  |  |  |  |  |  | 6 | 10.1 | 0.3 |
|  |  |  |  |  |  |  |  |  | 7 | 3.5 | 0.6 |
|  | Mean | 140,000 | 380 | 1.4 | 1.32 | 0.61 | 0.71 | 0.54 | 1 | 28.3 | -1.9 |
|  |  |  |  |  |  |  |  |  | 2 | 6.9 | 1.0 |
|  |  |  |  |  |  |  |  |  | 3 | 28.4 | -3.4 |
|  |  |  |  |  |  |  |  |  | 4 | 12.4 | -1.9 |
|  |  |  |  |  |  |  |  |  | 5 | 3.4 | -4.5 |
|  |  |  |  |  |  |  |  |  | 6 | 28.4 | -3.4 |
|  |  |  |  |  |  |  |  |  | 7 | 27.9 | -0.6 |
| *SV 2020* | May | 100,000 | 365 | 3.69 | 5.65 | 3.29 | 2.36 | 0.42 | 2 | 9.6 | 0.5 |
|  |  |  |  |  |  |  |  |  | 3 | 9.0 | -0.5 |
|  |  |  |  |  |  |  |  |  | 5 | 2.4 | -0.8 |
|  |  |  |  |  |  |  |  |  | 6 | 28.0 | -0.2 |
|  | June | 100,000 | 365 | 3.36 | 5.56 | 3.41 | 2.15 | 0.39 | 3 | 4.1 | 0.4 |
|  |  |  |  |  |  |  |  |  | 5 | 9.9 | 3.4 |
|  |  |  |  |  |  |  |  |  | 6 | 25.6 | 4.2 |
|  | July | 100,000 | 369 | 4.91 | 5.06 | 3.08 | 1.98 | 0.39 | 3 | 26.9 | 2.4 |
|  |  |  |  |  |  |  |  |  | 5 | 9.7 | 0.1 |
|  |  |  |  |  |  |  |  |  | 6 | 27.7 | 0.8 |
|  |  |  |  |  |  |  |  |  | 7 | 10.3 | 0.8 |
|  | Aug. | 100,000 | 367 | 2.51 | 2.81 | 2.04 | 0.77 | 0.27 | 3 | 6.0 | 2.8 |
|  |  |  |  |  |  |  |  |  | 6 | 26.9 | 2.1 |
|  | Nov. | 100,000 | 368 | 1.50 | 2.24 | 1.64 | 0.60 | 0.27 | 3 | 6.5 | 0.1 |
|  |  |  |  |  |  |  |  |  | 6 | 27.5 | 1.3 |
|  | Mean | 591,000 | 369 | 3.18 | 2.19 | 1.00 | 1.19 | 0.54 | 3 | 8.8 | 0.9 |
|  |  |  |  |  |  |  |  |  | 5 | 6.2 | 1.2 |
|  |  |  |  |  |  |  |  |  | 6 | na | 10.0 |
| *SV 2021* | May | 100,000 | 365 | 0.63 | 0.76 | 0.53 | 0.22 | 0.29 | 1 | 5.5 | -0.3 |
|  |  |  |  |  |  |  |  |  | 2 | 12.9 | 0.0 |
|  |  |  |  |  |  |  |  |  | 4 | 3.1 | -1.7 |
|  |  |  |  |  |  |  |  |  | 7 | 4.1 | 0.2 |
|  | June | 100,000 | 365 | 2.82 | 6.17 | 3.77 | 2.4 | 0.39 | 6 | 26.9 | 2.3 |
|  |  |  |  |  |  |  |  |  | 7 | 6.9 | 0.7 |
|  | July | 100,000 | 365 | 0.91 | 1.62 | 0.92 | 0.70 | 0.43 | 2 | 8.5 | 0.3 |
|  |  |  |  |  |  |  |  |  | 3 | 27.2 | 1.9 |
|  |  |  |  |  |  |  |  |  | 6 | 14.0 | 0.5 |
|  |  |  |  |  |  |  |  |  | 7 | 25.9 | 3.4 |
|  | Aug. | 100,000 | 365 | 0.98 | 1.77 | 1.17 | 0.60 | 0.34 | 2 | 4.3 | 0.8 |
|  |  |  |  |  |  |  |  |  | 3 | 10.2 | 0.7 |
|  | Oct. | 100,000 | 365 | 0.96 | 1.42 | 0.88 | 0.54 | 0.38 | 3 | 13.5 | 1.5 |
|  |  |  |  |  |  |  |  |  | 5 | 27.3 | -0.2 |
|  |  |  |  |  |  |  |  |  | 6 | 26.5 | 3.2 |
|  |  |  |  |  |  |  |  |  | 7 | 9.8 | 0.3 |
|  | Nov. | 100,000 | 365 | 2.79 | 4.56 | 2.47 | 2.09 | 0.46 | 3 | 27.2 | 2.3 |
|  |  |  |  |  |  |  |  |  | 5 | 13.6 | -0.8 |
|  |  |  |  |  |  |  |  |  | 6 | 28.2 | -1.0 |
|  |  |  |  |  |  |  |  |  | 7 | 27.0 | 1.5 |
|  | Mean | 100,000 | 365 | 1.5 | 1.26 | 0.53 | 0.73 | 0.58 | 3 | 27.6 | 1.1 |
|  |  |  |  |  |  |  |  |  | 5 | 27.4 | -0.4 |
|  |  |  |  |  |  |  |  |  | 6 | 28.1 | -0.6 |
|  |  |  |  |  |  |  |  |  | 7 | 27.0 | 1.7 |
| Markov chain Monte Carlo (MCMC) run length, phenotypic mean (*μ*), phenotypic variance (*σ^2^_P_*), residual variance(*σ^2^_e_*), additive variance(*σ^2^_A_*), narrow-sense heritability (*h^2^*), the linkage groups (LG) on which QTLs were mapped.  *2ln(BF)*. Bayes Factor, a measure quantifies the support from the data for the number of QTL(s) in the model (QTL evidence), after pair-wise model comparison (1/0, 2/1, and 3/2) such as ‘one-QTL model’ vs. ‘zero-QTL model, etc. *2ln(BF)* <0 = no evidence; 0-2 = hardly any; 2-5 = positive; 5-10 = strong; >10 = decisive. Bayes Factor will not be available (na) if either model has insufficient samples in the Markov chain. | | | | | | | | | | | |

| **Supplementary Table 12**. SNP name, genetic position (cM), and physical location for SNPs in each allele sequence of haplotypes identified for cercospora leaf spot (CLS) disease for nine rose breeding parents of the TX2WOB population. | | | | | | | | | | | |
| --- | --- | --- | --- | --- | --- | --- | --- | --- | --- | --- | --- |
| SNP name | Genetic position (cM) | Physical location | Haplotype | | | | | | | | |
| *q*CLS.TX2WOB-LG3.2 | | | A1 | A2 | A3 | A4 |  |  |  |  |  |
| chr03_18884374 | 25.4 | 18,884,374 | A | T | A | A |  |  |  |  |  |
| chr03_21408083 | 28.8 | 21,408,083 | A | G | G | A |  |  |  |  |  |
| chr03_22108249 | 31.7 | 22,108,249 | A | C | C | A |  |  |  |  |  |
| chr03_22061915 | 31.7 | 22,061,915 | A | C | C | A |  |  |  |  |  |
| chr03_22294302 | 33.0 | 22,294,302 | C | G | G | C |  |  |  |  |  |
| chr03_22634715 | 33.7 | 22,634,715 | G | T | T | - |  |  |  |  |  |
| chr03_22810215 | 34.3 | 22,810,215 | C | T | T | - |  |  |  |  |  |
| chr03_23490623 | 35.5 | 23,490,623 | T | G | G | T |  |  |  |  |  |
| chr03_23490556 | 35.5 | 23,490,556 | G | A | A | G |  |  |  |  |  |
| *q*CLS.TX2WOB-LG4.2 | | | C1 | C2 | C3 | C4 | C5 | C6 | C7 | C8 | C9 |
| chr04_35259242 | 34.1 | 35,259,242 | C | G | G | G | G | G | G | G | G |
| chr04_35707566 | 34.2 | 35,707,566 | T | A | T | A | A | T | T | T | T |
| chr04_35672064 | 34.6 | 35,672,064 | C | T | T | T | - | T | T | T | T |
| chr04_35721231 | 34.9 | 35,721,231 | C | A | A | A | A | C | A | C | C |
| chr04_35821774 | 35.2 | 35,821,774 | A | A | G | A | A | A | A | A | A |
| chr04_36699772 | 35.5 | 36,699,772 | A | A | G | A | A | A | A | G | G |
| chr04_38053715 | 35.6 | 38,053,715 | G | G | T | G | G | T | G | G | T |
| chr04_39709868 | 36.8 | 39,709,868 | G | G | G | - | G | C | G | C | G |

| **Supplementary Table 13**. SNP name, genetic position (cM), and physical location for SNPs in each allele sequence of haplotypes identified for cercospora leaf spot (CLS) disease for nine rose breeding parents of the TX2WSE population. | | | | | | | |
| --- | --- | --- | --- | --- | --- | --- | --- |
| SNP name | Genetic position (cM) | Physical location | Haplotype | | | | |
| *q*CLS.TX2WSE-LG3.1 | | | B1 | B2 | B3 | B4 | B5 |
| chr03_21519631 | 16.31 | 21,519,631 | T | C | C | C | C |
| chr03_18044384 | 16.88 | 18,044,384 | C | C | T | C | T |
| chr03_22902932 | 17.21 | 22,902,932 | T | T | A | A | T |
| chr03_22894266 | 17.74 | 22,894,266 | T | C | T | T | C |
| chr03_23443956 | 17.76 | 23,443,956 | A | A | G | G | A |
| chr03_27802201 | 18.39 | 27,802,201 | G | A | A | A | A |
| *q*CLS.TX2WSE-LG6.2 | | | D1 | D2 | D3 | D4 | D5 |
| chr06_17921337 | 31.88 | 17,921,337 | T | G | T | T | T |
| chr06_29543291 | 34.36 | 29,543,291 | G | A | G | A | G |
| chr06_28564363 | 35.24 | 28,564,363 | A | G | A | A | A |
| chr06_31682107 | 35.28 | 31,682,107 | T | C | T | T | - |
| chr06_29498481 | 36.31 | 29,498,481 | C | A | C | C | C |
| chr06_33885226 | 36.58 | 33,885,226 | C | T | C | C | C |
| chr06_33617397 | 36.84 | 33,617,397 | C | G | G | C | G |
